# Comparative analysis of the non-volatile metabolites and taste profiles of the four famous freshwater fish raw materials in China

**DOI:** 10.1016/j.fochx.2026.103623

**Published:** 2026-01-30

**Authors:** Lili Chen, Chaochao Wang, Yu Liu, Yongcheng Wang, Chunqing Bai, Yong Jiang, Meilan Yuan, Li Zhao, Renjie Wang

**Affiliations:** a School of Life Science, Jiangxi Science & Technology Normal University, Nanchang 330013, China; b Jiangxi engineering research center of aquatic product processing and safety control, Jiangxi Science&Technology Normal University, Nanchang 330013, China; c Jiangxi Province Key Laboratory of Organic Functional Molecules; Institute of Organic Chemistry, Jiangxi Science & Technology Normal University, Nanchang 330013, China

**Keywords:** Four famous freshwater fish, Metabolomics, Amino acids, Nucleotides, UPLC-MS, *E*-tongue

## Abstract

Four famous freshwater fish in China are renowned for their high production among freshwater species and are much-prized as sought-after delicacies. However, the distinct taste profiles and the specific associated taste substance of these carps remain poorly understood. This study primarily investigated the taste profiles and associated taste compounds, along with non- volatile metabolites of these four species using ultra performance liquid chromatography-mass spectrometry (UPLC-MS) combined with electronic tongue technology. Results revealed that *Mylopharyngodon piceus* (MP) exhibited significantly higher levels of total free amino acids and adenosine triphosphate (ATP) compared to the other three fish species (*p* < 0.05), with contents reaching approximately 388.90 mg/100 g and 140.88 mg/100 g, respectively. *Ctenopharyngodon idella* (CI) and MP possessed richer tastes compared to *Aristichthys nobilis* (AN) and *Hypophthalmichthys molitrix* (HM) based on the electronic tongue analysis. Furthermore, UPLC-MS metabolomics analysis identified 49 key metabolites, highlighting major metabolic pathways including lipid, amino acid, and organic acid metabolism. The findings suggested that variations in taste-related compounds and metabolites within metabolic pathways contribute to the distinct flavor profiles observed among the four famous fish species. This research offers essential insights for aquaculture professionals and researchers focused on enhancing the flavor quality of these key fish species, especially in understanding the regulatory mechanisms involved in flavor development

## Highlights

• UPLC-MS analysis identified 49 key metabolites in the four famous freshwater fish.
• Key metabolites in four fish species are mainly derived from the pathways of free amino acid metabolism, lipid metabolism and amino acid metabolism.
• *Ctenopharyngodon idella* (CI) and *Mylopharyngodon piceus* (MP) potentially possessed more pronounced taste flavors than *Aristichthys nobilis* (AN) and *Hypophthalmichthys molitrix* (HM).
• MP display the highest content of the total free amino acid at 388.90 mg/100 g.

## Graphical abstract

**Figure:**
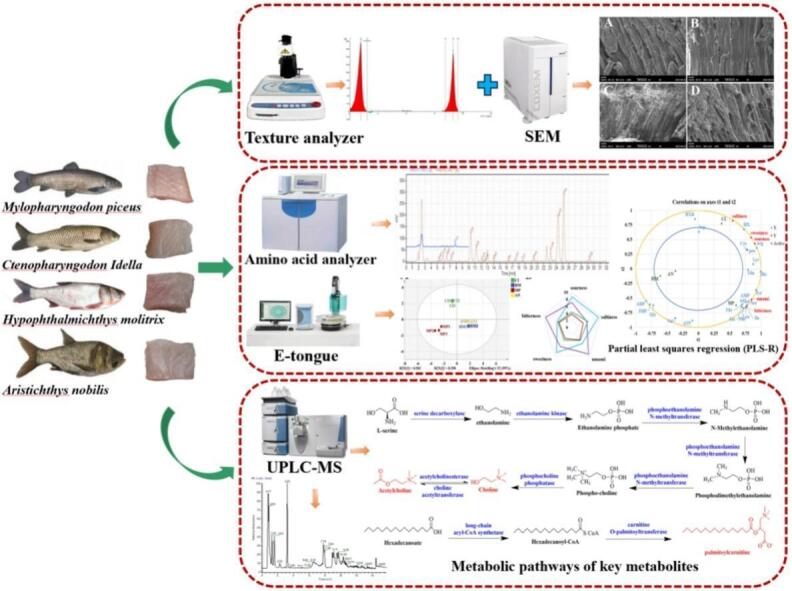

## 1 Introduction

Meat is a vital food for human beings, which can provide rich nutrients such as proteins and fats to fulfill the nutritional needs of human beings (Cui et al., 2023). Fish is characterized by high protein content and low fat composition (Jiang et al., 2022), which makes it popular among the groups with weight control requirement. *Ctenopharyngodon Idella* (CI), *Hypophthalmichthys molitrix* (HM), *Mylopharyngodon piceus* (MP) and *Aristichthys nobilis* (AN) are collectively known as the four famous freshwater fish in China. These four freshwater fish species serve primarily as regional food sources and are widely consumed throughout East and Southeast Asia, particularly in China, Vietnam, Thailand and neighbouring regions. They are the mainstay of freshwater aquaculture in the country, and their production makes up nearly half of China's freshwater fish production (according to the China Fishery Year Book, 2024). Since they are fairly priced and the meat is tasty, they are a common and delicious dish in Chinese households.

Research on four famous freshwater fish in China in the national series has primarily concentrated on CI, which boasts the highest annual production, a relatively short culture cycle, and low input costs. AN and HM follow in research focus, while MP have received less attention, primarily due to their longer culture cycles, higher input costs, and elevated market prices. Currently, studies on the quality of freshwater fish predominantly examine how culture methods, processing techniques, storage conditions, and handling practices impact fish quality. For instance, Zhang, Li, et al. (2016) investigated the influence of four different culture methods on the flavor and nutritional value of grass carp, revealing that grass carp raised on fava beans had superior qualities compared to those cultivated using other methods, including grass meal treatment, wave water culture, and commercial feed. Additionally, Li, Fan, et al. (2021) found that micro- and nano-starch significantly affected the gel properties of myofibrillar protein in grass carp. Zhang, Luo, et al. (2016) also explored how different stunning techniques impacted the stress levels and quality of silver carp fillets stored at 4 °C for 72 h. Furthermore, Yi et al. (2020) compared the gel properties and flavor profiles between anchovy minced meat and silver carp minced meat. To the best of our knowledge, there has yet to be any research published on the taste profiles and metabolite analysis of four famous freshwater fish in China.

Taste of fish is closely related to its non-volatile compounds, plays a significant role in influencing human food preferences. Electronic tongue (*E*-tongue) is a smart analytical instrument that simulates human taste, enabling the scientific and objective assessment of sensory properties while mitigating the inherent subjectivity associated with human sensory evaluation (Lu et al., 2022). Metabolomics is a validated analytical method for the analysis of small molecule endogenous metabolites identified. In recent years, there has been an increasingly utilization of metabolomics approaches in various area such as food processing, food testing, food security, etc. (Wu et al., 2022), especially in detecting the intrinsic mechanisms of food quality and its metabolic pathways. For instance, Liu, Wang, et al. (2024) characterized the flavor of citrus peels from different aging years by sensory and metabolomic approaches. Liu et al. (2022) revealed the changes of small molecules in duck breast under different preservation times by non-targeted metabolomics. Non-targeted metabolomics can analyze as many small molecule metabolites as possible, and can be preferred when there is uncertainty about the pathway of a metabolic species, as this methodology helps to differentiate small differences in scientific research (Ribbenstedt et al., 2018).

In our previous research, we comparatively analyzed the differences in volatile flavors of the four major Chinese carps (Wang et al., 2025). Actually, non-volatile compounds also play a crucial role in the overall taste of a food. Therefore, we used an *E*-tongue to compare the taste differences among the four major fish species. The potential metabolite differences among the four fish species and their relationship with taste presentation were explored by quantitative analysis of free amino acids and nucleotides and their derivatives, as well as identification of small molecule metabolites using UPLC-MS. Additionally, the texture and structural dissimilarities among these four fish species were analyzed through texture analysis and scanning electron microscopy. The results serve as a crucial guidance for improving the textural and taste attributes, as well as assessing, processing, and optimizing the culinary applications of these four prominent freshwater fish species in China.

## 2 Material and methods

### 2.1 Samples

**Fig. 1:**
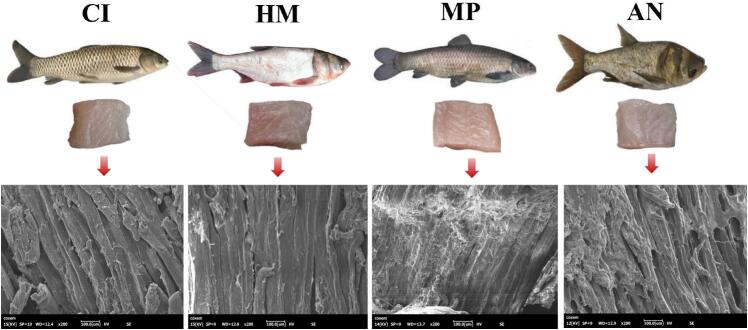
Diagram of full carps and microstructure of four famous freshwater fish in China by scanning electron microscopy (SEM). Note: CI: *Ctenopharyngodon Idella*, HM: *Hypophthalmichthys molitrix*, MP: *Mylopharyngodon piceus*, AN: *Aristichthys nobilis*.

Four well-known freshwater fish species (three individuals of each species) in China were obtained from Jinxian County Yanhu Special Aquatic Products Development Company, located in Nanchang, Jiangxi Province (see Fig. 1). The fish were transported alive to the aquatic products processing laboratory in oxygenated containers under controlled temperature conditions (approximately 18–20 °C), and all samples were processed within 2 h after arrival to minimize post-mortem biochemical changes. Upon arrival, the fish were immediately processed by removing the head, scales, and skin, and only the edible muscle portions were retained. These portions were then sectioned and stored at −80 °C for preservation. As detailed in our previous study (Wang et al., 2025), the fundamental nutritional composition and several physicochemical properties of the samples were analyzed.

### 2.2 Texture measurement

Cut the meat from the back of four famous freshwater fish in China into pieces measuring 2 cm × 2 cm × 1 cm. The texture of the fish pieces was analyzed using a texture analyzer (CT3–4500, Brookfield Corporation, USA) to measure springiness, hardness, gumminess, cohesiveness, and chewiness. The measurement conditions were set according to Gao, Liu, et al. (2023) with minor modifications as follows: Texture Profile Analysis (TPA) was used as the experimental mode, a 5 mm diameter stainless steel cylindrical probe was utilized, and the initial, test, and probe's post-test speed was configured to 1 mm/s with a trigger point load of 5 g and compression cycle repeated twice. For each fish species, three independent biological samples (*n* = 3) were collected. Each biological sample was analyzed in duplicate as technical replicates to assess analytical reproducibility, resulting in six technical measurements per species.

### 2.3 Performing microstructure analysis by employing scanning Electron microscopy (SEM)

We adopted the method from Lin et al. (2022) and made slight modifications to it during the application. Initially, the samples were immersed in a 2.5% glutaraldehyde solution for 5 h. Following this, they underwent three times rinsed using a pH 7.0, 0.1 mol/L phosphate buffer solution, with each rinse lasting for 15 min. After that, a dehydration process was carried out on the samples. Ethanol solutions at different concentrations, namely 30%, 50%, 70%, 80%, 90%, 95%, and 100%, were applied successively, with each concentration being kept in contact with the samples for 15 min. Subsequently, the samples were immersed in a 1:1 ethanol-tert-butanol solution for 15 min and then in tert-butanol alone for an additional 15 min. Eventually, the samples were placed in storage at a temperature of −70 °C for the purpose of freezing. The lyophilized samples were then gold-coated and inspected via a scanning electron microscope (COXEM EM-30PLUS, Korea).

### 2.4 Determination of free amino acids (FAAs)

The free amino acids (FAAs) contents of four famous freshwater fish in China were measured based on the method described by Zhu et al. (2023) with some modifications. Accurately weigh the sample (1.0 g) was mechanically disrupted with 15 mL Trichloroacetic acid (15%) for 2 min. After sitting for two hours, the sample solution was centrifuged (4 °C, 10,000 r/min, 15 min). Take 5 mL of supernatant, adjust pH to 2.0 with NaOH (3 mol/L), volume to 10 mL and filtered through a 0.22 μm filter. Quantification of FAAs was performed via an automatic amino acid analyzer (L-8080, Hitachi, Tokyo, Japan). Three replicate measurements were carried out.

### 2.5 Determination of nucleotides and their derivatives

The method was adjusted slightly in line with Xiao et al. (2021). Specifically, a fish sample with a mass of 5 *g* was moved to a centrifuge tube, and then 10 mL of 10% cold perchloric acid was added thereto. Following 2 min of homogenization, the mixture was centrifuged at 10,000 rpm and 4 °C for 15 min to obtain the supernatant. The precipitate was washed by adding an additional 5 mL of 5% cold perchloric acid, followed by centrifugation under the same conditions. Twice was the washing step repeated. After that, the resulting supernatants were put together. And the pH of the combined supernatant was modified to 7.0 with the help of 10 mol/L or 1 mol/L KOH. The solution was then allowed to stand in a refrigerator at 4 °C for 30 min. Subsequently, the solution was reconstituted to a final volume of 50 mL, thoroughly mixed, and filtered through a 0.22 μm membrane prior to instrumental analysis.

HPLC Experimental Parameters: An Agilent 5TC-C18 (2) column (250 × 4.6 mm) was utilized for Arc HPLC (Waters, USA) under the following conditions: 35 °C was maintained as the column temperature, 254 nm was set for the detection wavelength, 1 mL/min was adopted as the flow rate, and 10 μL was designated as the injection volume. The mobile phase consisted of two components: Phase A was a 0.5 mol/L KH_2_PO_4_-K_2_HPO_4_ buffer solution, with the pH adjusted to 7.0 using phosphoric acid, while Phase B was a solution made from methanol. An isoconcentration gradient was utilized to conduct the elution.

Preparation of standard curves for nucleotides and their derivatives: Standard curves for adenosine-5′-triphosphate (ATP), adenosine-5′-diphosphate (ADP), adenosine-5′-monophosphate (AMP), inosine monophosphate (IMP), and hypoxanthine ribonucleoside (HxR) were prepared using concentrations of 0.200 μg/mL, 0.500 μg/mL, 1.000 μg/mL, 5.00 μg/mL, 15.0 μg/mL, 40.0 μg/mL, and 100 μg/mL. For hyperxanthine (Hx), concentrations of 0.100 μg/mL, 0.250 μg/mL, 0.500 μg/mL, 7.50 μg/mL, 20.0 μg/mL, and 50.0 μg/mL were used. The standard curves were plotted under the same conditions as those used for sample determination, as detailed in Table S1.

### 2.6 Electronic tongue analysis

The taste attributes of four famous freshwater fish in China were analyzed using the Astree electric tongue (Alpha MOS, France), which consisted of an automatic sampling system, a sensor array (comprising 7 sensors including AHS, PKS, CTS, NMS, CPS, ANS, SCS), and a data processing system. Electronic tongue analysis was conducted according to the method described by Park and Kim (2021), with minor modifications. In brief, a mixture of 20 g fish and 50 g water were blended for 30 s to obtain a homogenate, and after centrifugation (1500 rpm, 15 min in ordinary temperature), 2.0 g of supernatant was extracted and diluted tenfold before measurement.

### 2.7 Extraction of metabolites from fish

Fish tissue samples were prepared based on a previously described method by Chen, Hong, et al. (2023),with minor modifications. Approximately 50 mg of fish muscle tissue was weighed into a 2 mL centrifuge tube. Then, 800 μL of 80% methanol was added, and the mixture was vortexed and shaken thoroughly. The sample was ground at 65 Hz for 180 s and sonicated at 4 °C for 30 min. It was then left at −40 °C for 1 h, followed by 30 s of vortexing and a further 30 min at 4 °C. Centrifuge the sample at 12,000 rpm for 15 min at 4 °C. Following this, collect the supernatant into a new centrifuge tube. Finally, keep it at −40 °C for 1 h. A second centrifugation was performed at 4 °C for 15 min 12,000 rpm. 200 μL was pipetted from the supernatant, and then 5 μL of internal standard (0.20 mg/mL dichlorophenylalanine) was added to it. This mixture was thoroughly mixed and transferred to the injection vial. Additionally, a pooled quality control (QC) sample was prepared by mixing equal aliquots of extracts from all experimental samples. The QC sample was used to monitor the stability, reproducibility, and analytical performance of the analytical platform throughout the entire analytical run.

### 2.8 Metabolites analysis by UPLC-MS

The instrumental platform utilized for this analysis was LC-MS (Waters, UPLC; Thermo, Q Exactive), and the chromatographic column employed was ACQUITY UPLC HSS T3 (2.1 × 100 mm 1.8 μm). The chromatographic and mass spectrometric conditions were established with reference to the method described by Yu et al. (2022), with appropriate modifications made to suit the experimental requirements. The column temperature was fixed at 40 °C, and the flow rate was held at 0.300 mL/min. The injection volume was 5 μL, and the automatic injector temperature was maintained at 4 °C. In both ESI+ and ESI- modes, the mobile phase consists of water with 0.05% ammonium as component A, and acetonitrile as component B. The gradient elution procedure for the mobile phase was carried out as follows: From 0 to 1 min, component A was maintained at 95% and component B at 5%. From 1 to 12.5 min, component A decreased linearly from 95% to 5%, while component B increased linearly from 5% to 95%, and this ratio was held for 1 min. Between 13.5 and 13.6 min, component A transitioned linearly from 5% to 95% and component B from 95% to 5%. Finally, from 13.6 to 16 min, component A was held at 95% and component B at 5%.

The Q Exactive high-resolution mass spectrometer (Thermo Fisher Scientific) was used for mass spectrometry analysis. The mass spectrometry detection parameters are as follows: In the ESI+ mode, the heater temperature was set to 300 °C. The sheath gas, auxiliary gas, and sweep gas flow rates were maintained at 45, 15, and 1 arb, respectively, for both ESI+ and ESI- modes. The capillary temperature was fixed at 350 °C in both modes. In ESI+ mode, the spray voltage was set to 3.0 kV, S-Lens RF level to 30%, and heater temperature to 300 °C. For ESI- mode, only the spray voltage (3.2 kV) and S-Lens RF level (60%) were adjusted, while other parameters remained unchanged.

Scanning modes include a primary full scan covering a mass-to-charge ratio (*m/z*) range of 70 to 1050, as well as a data-dependent secondary mass spectrometry scan (dd-MS2) targeting the top 10 ions. The resolution is set at 70,000 for the primary mass spectrometry scan and 17,500 for the secondary scan. The collision mode employed is high energy collisional dissociation (HCD).

### 2.9 Statistical analysis

All experiments were performed three times (*n* = 3), with the outcomes presented as mean values ± standard deviation. Differences among species were evaluated using one-way analysis of variance (ANOVA), with the significance level set at *P* < 0.05. SPSS 26.0 (SPSS Inc., Chicago, IL, USA) was utilized for experimental data analysis, while graphs were generated using Origin2021 (Origin, Inc., Massachusetts, USA). Non-volatile compounds were assessed through principal components analysis (PCA) and orthogonal partial least squares discriminant analysis (OPLS-DA) using SIMCA 14.1 (Umetrics, Umeå, Sweden), with key compounds identified based on a variable importance in the project (VIP) score greater than 1.5. Subsequently, the metabolic pathways of these key metabolites were analyzed by KEGG.

## 3 Result and discussion

### 3.1 Analysis of textural properties

The texture of fish meat differs significantly from that of other meats, and its textural properties being crucial for evaluating the quality of fishery products (Fu et al., 2024). According to Table 1, CI exhibited superior textural properties compared to the other three species. Previous research had revealed that CI had a lower moisture content than the other species, which was consistent with Gao, Qiao, et al. (2023) findings that lower moisture content was associated with higher textural properties. Springiness and cohesiveness showed no significant differences among four famous freshwater fish in China (*p* > 0.05). CI and HM exhibited significantly higher hardness values than MP and AN (*p* < 0.05). According to Boughattas et al. (2020), the hardness of fish muscles may be linked to amino acid composition. Gumminess is defined as the result of cohesiveness multiplied by hardness, indicating the ability of the material to remain intact and cohesive when chewed, A higher level of gumminess corresponds to a better taste in carp (Gao, Qiao, et al., 2023). In CI, the value of gumminess was markedly higher compared to the other three fish species, indicating better taste in CI comparably. Chewiness, an overall assessment of food texture combining hardness, springiness, and cohesiveness, was highest in CI. Conversely, the chewiness of MP and AN was notably lower (*p* < 0.05), indicating potential tenderness in their meat compared to the tougher texture of CI and HM.

**Table 1:** Textural parameter of four famous freshwater fish in China.

	**CI**	**HM**	**MP**	**AN**
**Hardness (g)**	208.03 ± 13.82^a^	192.97 ± 3.34^a^	89.05 ± 1.75^b^	97.16 ± 0.90^b^
**Springiness**	0.75 ± 0.03^a^	0.72 ± 0.05^a^	0.68 ± 0.02^a^	0.68 ± 0.03^a^
**Cohesiveness**	0.66 ± 0.06^a^	0.66 ± 0.04^a^	0.67 ± 0.01^a^	0.66 ± 0.03^a^
**Gumminess (g)**	137.22 ± 5.56^a^	126.65 ± 5.74^b^	59.43 ± 1.68^c^	64.12 ± 1.60^c^
**Chewiness (g)**	105.51 ± 5.43^a^	87.05 ± 5.82^b^	40.56 ± 0.42^c^	43.90 ± 2.40^c^

**Note:** CI: *Ctenopharyngodon Idella*, HM: *Hypophthalmichthys molitrix*, MP: *Mylopharyngodon piceus*, AN: *Aristichthys nobilis*. The value is expressed as S.D. ± mean. Significant differences are represented by distinct letters within the same row (*p* < 0.05).

### 3.2 Analysis of microstructure

Muscle fiber plays a primary role in influencing the texture of fish meat, which visually impacts the tissue structure of the fish (Shi et al., 2020). differences in muscle fiber microstructures among the four fish species are depicted in Fig. 1, revealing general similarities. All four species exhibit long and thin strip-like muscle fibers with small and closely connected gaps. Research suggested that fish with lower moisture content tend to have smaller gaps between muscle fibers (Xia et al., 2023). Fig. 1 highlighted that AN display a more disorganized fibrous structure, possibly resulting from temperature fluctuations during the freeze-thaw process, leading to some degree of damage to the fish's muscle structure.

### 3.3 Analysis of free amino acids (FAAs)

To a certain extent, the types, composition and concentration of free amino acids contributed distinctive flavor profiles to fishery products (Luo et al., 2022). In this research, 17 FAAs were quantitatively determined in four carps. These amino acids can be categorized into umami amino acids (UAAs), sweet amino acids (SAAs) and bitter amino acids (BAAs) based on their taste characteristics. In general, FAAs such as glutamic acid, aspartic acid, alanine, and glycine are mainly related to umami and sweet tastes, whereas amino acids including leucine, isoleucine, valine, phenylalanine, and histidine are often associated with bitter taste (Min et al., 2023). Therefore, both the total FAA content and the relative composition of individual amino acids jointly influence the overall taste profile of fish muscle (Lee et al., 2022). As shown in Table 2, the total concentration in CI, HM, MP, AN group was 292.50, 214.50, 388.90, 228.45 mg/100 g, respectively. Notably, in contrast to the other three groups, the MP group had levels that were substantially (*p* < 0.05) higher. The differences in FAAs content among four well-known freshwater fish species account for differences in taste. It has been reported that amino acids affect the sensors of the electronic tongue, leading to alterations in taste values (Cai et al., 2024).

**Table 2:** the content of free amino acids (FAAs) in four famous freshwater fish in China.

Free amino acids	Threshold (mg/100 g)	Concentration in four famous freshwater fish in China (mg/100 g)			
		CI	HM	MP	AN
Asp	3	0.58 ± 0.35^a^	0.54 ± 0.42^a^	0.39 ± 0.45^a^	0.33 ± 0.50^a^
Glu	30	7.30 ± 1.04^a^	5.96 ± 1.21^a^	7.82 ± 0.93^a^	4.03 ± 3.68^a^
**ΣUAA**		7.87 ± 1.23^a^	6.51 ± 1.55^a^	8.20 ± 1.05^a^	4.37 ± 4.18^a^
Thr	260	8.70 ± 1.67^b^	6.71 ± 2.57^b^	14.77 ± 3.00^a^	10.60 ± 2.28^ab^
Arg	50	7.56 ± 2.59^a^	0.88 ± 0.13^b^	5.90 ± 3.79^ab^	1.51 ± 1.98^b^
Pro	300	12.08 ± 5.70^a^	7.48 ± 0.69^ab^	10.37 ± 3.57^ab^	4.42 ± 1.81^b^
Gly	130	30.54 ± 8.40^a^	62.62 ± 29.40^a^	46.07 ± 9.14^a^	47.49 ± 8.75^a^
Ala	60	23.71 ± 4.51^b^	18.74 ± 6.60^b^	43.67 ± 9.01^a^	29.68 ± 2.28^b^
Ser	150	3.59 ± 3.93^ab^	1.46 ± 1.40^b^	10.11 ± 4.37^a^	1.59 ± 1.96^b^
**ΣSAA**		86.20 ± 23.58^a^	97.89 ± 27.94^a^	130.88 ± 32.20^a^	95.29 ± 15.21^a^
Cys	–	0.30 ± 0.08^a^	0.20 ± 0.03^a^	0.26 ± 0.14^a^	0.13 ± 0.03^a^
Val	40	0.26 ± 0.09^a^	0.37 ± 0.37^a^	0.35 ± 0.10^a^	0.31 ± 0.12^a^
Met	30	2.30 ± 0.25^b^	1.46 ± 1.40^b^	5.51 ± 0.41^a^	2.44 ± 1.93^b^
Ile	90	1.72 ± 0.40^b^	0.66 ± 0.62^b^	7.66 ± 2.44^a^	1.70 ± 1.04^b^
Leu	190	3.64 ± 0.89^b^	2.13 ± 1.64^b^	13.45 ± 3.51^a^	3.81 ± 1.88^b^
Tyr	–	3.24 ± 0.16^ab^	1.58 ± 0.52^b^	5.04 ± 2.55^a^	2.66 ± 0.67^ab^
Phe	90	6.55 ± 0.41^b^	6.56 ± 4.59^b^	12.53 ± 1.37^a^	5.57 ± 0.68^b^
Lys	50	20.26 ± 6.93^a^	13.29 ± 5.62^a^	20.90 ± 8.55^a^	18.60 ± 3.64^a^
His	20	160.17 ± 35.89^a^	83.87 ± 7.88^b^	184.10 ± 8.21^a^	93.58 ± 23.55^b^
**ΣBAA**		198.44 ± 41.95^a^	110.11 ± 20.13^b^	249.80 ± 23.57^a^	128.79 ± 21.16^b^
**Total**		292.50 ± 60.16^b^	214.50 ± 14.55^b^	388.90 ± 56.27^a^	228.45 ± 32.50^b^

**Note:** CI: *Ctenopharyngodon Idella*, HM: *Hypophthalmichthys molitrix*, MP: *Mylopharyngodon piceus*, AN: *Aristichthys nobilis*. **ΣUAA** means the total of umami amino acids; **ΣSAA** means the total of sweet amino acids; **ΣBAA** means the total of bitter amino acids. The value is expressed as S.D. ± mean. Significant differences are indicated by different letters in the same row (*p < 0.05*)*.*

This could potentially account for the significant distinction observed between the MP group and the other three groups. What's more, the concentration of asparatic acid (Asp), glutamic acid (Glu), glycine (Gly), cysteine (Cys), valine (Val), and lysine (Lys) in the four fish had no significant difference (*p* > 0.05). Asp and Glu are primary UAAs found in fishery products, and it had been reported that both of them have strong synergistic effects with fresh flavor nucleotides (Ngapo & Vachon, 2016). Histidine (His) displayed the highest concentration in all four fish species, thereby imparting a meat – like taste characteristic to the fish (Qin et al., 2020). Furthermore, it is noteworthy that His was the sole amino acid detected beyond the threshold level, indicating its significant impact on fish flavor. The concentration of alanine (Ala), methionine (Met), isoleucine (Ile), leucine (Leu), phenylalanine (Phe) in MP was significantly higher than the other three fish species, and they are all BAAs except for Ala. It is possible that this is a vital factor contributing to the intensified bitterness intensity that was subsequently noticed in MP with the help of *E*-tongue analysis.

### 3.4 Analysis of nucleotides and their derivatives

Nucleotides and their derivatives play crucial physiological and biochemical roles in living organisms. Being the fundamental units of nucleic acid macromolecules, they play a crucial role in life, as stated by Ding et al. (2021). Additionally, they are key nourishing and flavor substances found in aquatic products (Xiao et al., 2021). After fish are slaughtered, ATP in the flesh is converted into ADP, AMP, IMP, Hx, and HxR (Liu et al., 2022). Among these, AMP and IMP are regarded as the primary flavor nucleotides in fish. IMP is the primary source of fresh flavor in meat products and serves as a key indicator of their freshness. Concurrently, IMP can react synergistically with glutamate and aspartate to produce an intense savoury flavor (Chen et al., 2022). AMP, as a potent flavor enhancer, effectively suppresses bitterness and imparts an excellent salty-sweet flavor to food (Dong et al., 2020). AMP and IMP are nucleotides that enhance taste perception by binding to the same T1R1 + T1R3 receptor as amino acids, thereby amplifying umami flavor (Bolumar et al., 2024). Moreover, the combined effect of IMP and AMP significantly enhances the freshness of the fish. HxR and Hx indicate the spoilage of fish, with higher values corresponding to greater levels of spoilage (Dong et al., 2020). Notably, Hx is associated with an unpleasant bitter flavor, the accumulation of which leads to a deterioration in the flavor of fish. With the aim of analyzing the distinct flavor profiles of four famous freshwater fish in China, we investigated their nucleotides and derivatives.

**Fig. 2:**
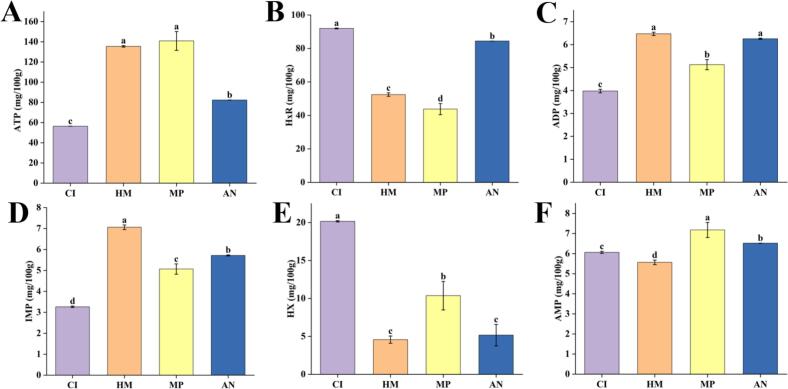
The content of nucleotides and their derivatives in four famous freshwater fish in China: ATP (A); HxR (B); ADP (C); IMP (D); Hx (E); AMP (F). **Note:** CI: *Ctenopharyngodon Idella*, HM: *Hypophthalmichthys molitrix*, MP: *Mylopharyngodon piceus*, AN: *Aristichthys nobilis*. *Different letters above bars indicate significant differences among species (p < 0.05).*

It can be seen from Fig. 2A that the ATP content in MP and HM was notably higher (*p* < 0.05) compared to that in AN and CI. It is hypothesized that the rate of ATP conversion in the flesh of AN and CI after slaughter is faster than in MP and HM. Additionally, the levels of HxR and Hx in CI were significantly increased (*p* < 0.05) compared to those in the other three species (Fig. 2B and Fig. 2E), indicating that CI may spoil more rapidly than that of the other species after slaughter. The levels of IMP and AMP varied significantly (*p* < 0.05) among the four main fish species. HM exhibited a notably higher content of IMP (Fig. 2D), suggesting a strong fresh flavor, while MP demonstrated a significantly elevated AMP level (Fig. 2F), indicating an enhanced ability to suppress bitter taste. Overall, HM and MP exhibited greater freshness, whereas CI was more prone to spoilage after slaughter in comparison.

### 3.5 Analysis of *E*-tongue

**Fig. 3:**
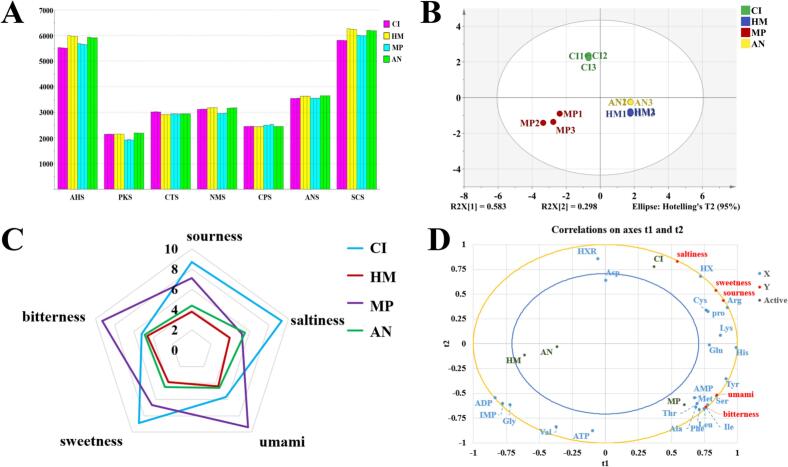
Analysis of E-tongue and partial least squares regression (PLS-R) analysis: Response intensity values of electronic tongue sensors in four famous freshwater fish in China (A); Principal component analysis (PCA) of four famous freshwater fish in China (B); Radar map of the taste attributes scores in four famous freshwater fish in China (C); Partial least squares regression (PLS-R) plot shows the relationship between the chemical composition and sensory attribute of four famous freshwater fish in China (D). **Note:** CI: *Ctenopharyngodon Idella*, HM: *Hypophthalmichthys molitrix*, MP: *Mylopharyngodon piceus*, AN: *Aristichthys nobilis*.

The E-tongue, which is designed for taste analysis, functions based on principles analogous to those of the human tongue, yet it gets rid of the subjective aspect of human sensory evaluation (Chen, Ning, et al., 2023). The *E*-tongue provides exceptional sensitivity, rapid detection capabilities, and low detection limits for food safety indicators, establishing itself as an invaluable tool for quality monitoring across industries such as food, cosmetics, and pharmaceuticals. The response values of the electronic tongue sensors are directly linked to the flavor intensity of the identified food (Zhang, Luo, et al., 2016). Table S2 displays the response values of the seven sensors for the four primary fish types, showcasing their high accuracy with relative standard deviations (RSDs) below 1%. In Fig. 3A, the sensors exhibit significant difference in intensity across different samples while demonstrating excellent repeatability within the same sample.

In the PCA plot of the *E*-tongue (Fig. 3B), the combined contributions of PC1 (58.3%) and PC2 (29.8%) amount to a total of 88.1%, effectively capturing and reflecting the samples' distinctive characteristics. The data points for AN and HM were more distant from those of CI and MP on PC1, indicating greater taste differences between AN/HM and CI/MP. Conversely, the close proximity of AN and HM on the PCA plot suggested a similarity in their taste profiles.

Based on the obtained data, a radargram of the E-tongue (Fig.3C) was generated to illustrate the response of the four carp samples to sourness, saltiness, bitterness, sweetness, and umami, it was observed that HM exhibited the lowest values for all five tastes compared to the other three fish. Additionally, CI displayed significantly higher levels of sourness, saltiness, and sweetness than the other three fish. On the other hand, MP showed significantly higher of levels bitterness and umami compared to AN, CI, and HN. These findings indicated that CI and MP may possess richer tastes profiles in comparison with AN and HM. Consistent with the bitter amino acid differences observed in the four major fishes talked about above is the fact that the bitter amino acid content was remarkably higher in CI and MP as opposed to HM and AN. This finding thereby verifies such consistency. The findings indicated that the integration of an electronic tongue with principal component analysis (PCA) is effective for categorizing four major fishes. It should be emphasized that, unlike traditional analytical instruments, an electronic tongue does not provide the exact concentration of a specific component. Instead, it generates a comprehensive response signal reflecting the overall physicochemical characteristics of the sample solution. The primary objective is to enable the differentiation and recognition of the “taste” profiles of various samples at a macroscopic level.

### 3.6 Relationship between chemical composition and sensory quality of four famous freshwater fish in China

Taking 17 free amino acids and 6 nucleotides in the four major carp as explanatory variables (X), partial least squares regression analysis (PLS-R) was carried out with the intensity of 5 sensory attributes of umami, saltiness, bitterness, sweetness and sourness as dependent variables (Y) to study how the sensory attributes of four famous freshwater fish in China are correlated with the chemical components of free amino acids and nucleotides. As shown in Fig. 3D, each sensory attribute has a different degree of correlation with flavor compounds. AN and HM were clustered together in the third quadrant, while CI and MP were positioned in the first and fourth quadrants respectively, and t2 separated CI from the other three groups, indicating that CI and other three fish were quite different in taste attributes and chemical composition, which was similar to the previous analysis of electronic nose and volatile compounds, that is, CI was relatively different from the other three types of fish.

Umami and AMP exhibited a significant positive correlation (*r* = 0.821), while glutamic acid (Glu) had a relatively weak correlation with umami (*r* = 0.730), while aspartic acid (Asp) was negatively correlated with umami (*r* = −0.265). It should be highlighted that while both Glu and Asp possess a carboxyl group (–COOH) in their side chains and serve as key precursors for umami flavor, their differing impacts on umami taste may stem from the stronger synergistic interaction between glutamate and umami nucleotides like AMP. This combination binds to the T1R1/T1R3 umami receptor, forming an “umami-enhancing complex” that intensifies the perception of glutamate's umami taste (Zhang et al., 2008). On the other hand, Asp can be converted into other metabolites in fish meat through decarboxylation or transamination (Gao, Qiao, et al., 2023). In conclusion, umami perception was the result of the dynamic equilibrium of multiple compounds, rather than the linear action of a single amino acid or a single nucleotide. Furthermore, apart from taste-active nucleotides and free amino acids, peptides and organic acids that contribute to taste also play crucial roles in shaping the overall flavor characteristics of fish.

### 3.7 LC-MS/MS analysis

#### 3.7.1 Metabolomics analysis of four famous freshwater fish in China

LC-MS/MS technology was utilized to detect the metabolites of four famous freshwater fish in China in both positive (ESI+) and negative (ESI-) modes. As shown in Fig. S2, the Total Ion Characterization (TIC) chromatogram was presented. Specifically, a total of 24,006 peaks were retrieved in the positive ion mode, while 15,293 peaks were retrieved in the negative ion mode. Subsequently, after removing ambiguities and considering accurate mass, MS2 spectra, etc., a final count of 371 metabolites in positive mode (ESI+) and 501 metabolites in negative mode (ESI-) were ultimately ascertained, resulting in a total of 872 metabolites.

**Fig. 4:**
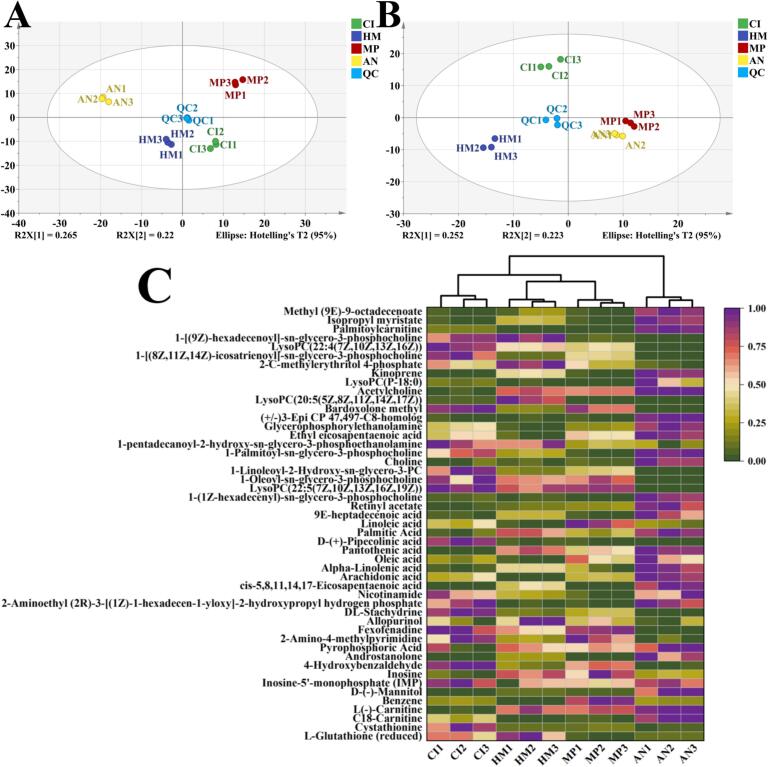
Analysis of nonvolatile compounds: Principal component analysis (PCA) of nonvolatile compounds in ESI- mode for four famous freshwater fish in China (A); Principal component analysis (PCA) of nonvolatile compounds in ESI+ mode for four famous freshwater fish in China (B); A heat map of the nonvolatile compounds that screening with VIP > 1.5 and *p* < 0.05 in four famous freshwater fish in China (C). **Note:** CI: *Ctenopharyngodon Idella*, HM: *Hypophthalmichthys molitrix*, MP: *Mylopharyngodon piceus*, AN: *Aristichthys nobilis*.

Fig. 4A-B respectively display the PCA results of the samples from the four carps and QC samples in ESI+ and ESI- modes. Specifically, in the ESI+ mode, the first two principal components could explain 47.5% of the total variance. In contrast, in the ESI- mode, they accounted for 48.7% of the total variance. It is worth noting that in both modes, all QC samples were closely grouped together at the center of the PCA scoring chart, indicating stability of data detection and reliability of instrumental analysis systems (Liu, Wen, et al., 2024). Differential metabolites identified were capable of reflecting non-volatile compositional differences among four famous freshwater fish in China (Li, Sun, et al., 2021). Moreover, distinct separation among all four fish species was observed through PCA in both ESI+ and ESI- modes, and each set of samples was well clustered. Result of PCA indicated that this method can partially differentiate the non-volatile metabolite differences among the four fish species.

A supervised multivariate data analysis model (OPLS-DA model) was established in order to maximize the differentiation among four famous freshwater fish in China in terms of metabolites, The scoring plot and twosided validation model were shown in Fig. S1. Each group of models showed good cluster separation. Table S2 listed the statistical parameters (R2X, R2Y, Q2) of OPLS-DA obtained from 200 validation tests. All results showed no overfitting of the OPLS-DA model, thereby indicating acceptable model robustness and stability. These results suggest that the OPLS-DA models effectively captured metabolic differences among the four fish species.

#### 3.7.2 Key metabolite screening and analysis

Projected variable importance (VIP) was obtained by developing a supervised multivariate data analysis (OPLS-DA) model. A total of 49 key metabolites (Table S4) was screened with VIP > 1.5 and *p* < 0.05, including 23 lipids and analogues, 9 organic acids, 4 amino acids and derivatives, 2 nucleotides, 6 organic nitrogen compounds, and 5 other metabolites. These metabolites are crucial for distinguishing the non-volatile differences among four famous freshwater fish in China. As shown in Fig. 4C, to visualize the similarities and differences in these metabolites across these four fish, cluster analysis was performed and plotted on a heat map. The results revealed significant differences between AN and the other three fish species; However, some degree of similarity was observed between HM and MP regarding their metabolite profiles.

The 49 identified differential metabolites were subjected to KEGG metabolic pathway analysis, which revealed a total of 8 metabolic pathways (Table S3). The findings suggested that these differential metabolites were predominantly associated with amino acid metabolism, lipid metabolism, organic acid metabolism and other associated processes.

##### 3.7.2.1 Lipids and analogues

Lipids, being an important substance in fish, have a decisive impact on the flavor and texture of the fish. Additionally, they also contribute to maintaining its tenderness and juiciness (Amaral et al., 2018). Lipids can be sorted into 3 main categories: fatty acyls (FAs), glycerides (GLs), and phospholipids (PLs). PLs can provide physiological needs of the organism during the forming of tissues and membrane-forming structures, while GLs can provide energy source for the organism, and FAs are essential for synthetizing GLs (Tian et al., 2024). The screened 23 key lipids included 8 GLs, 8 PLs, and 7 Fas (Table S4).

**Fig. 5:**
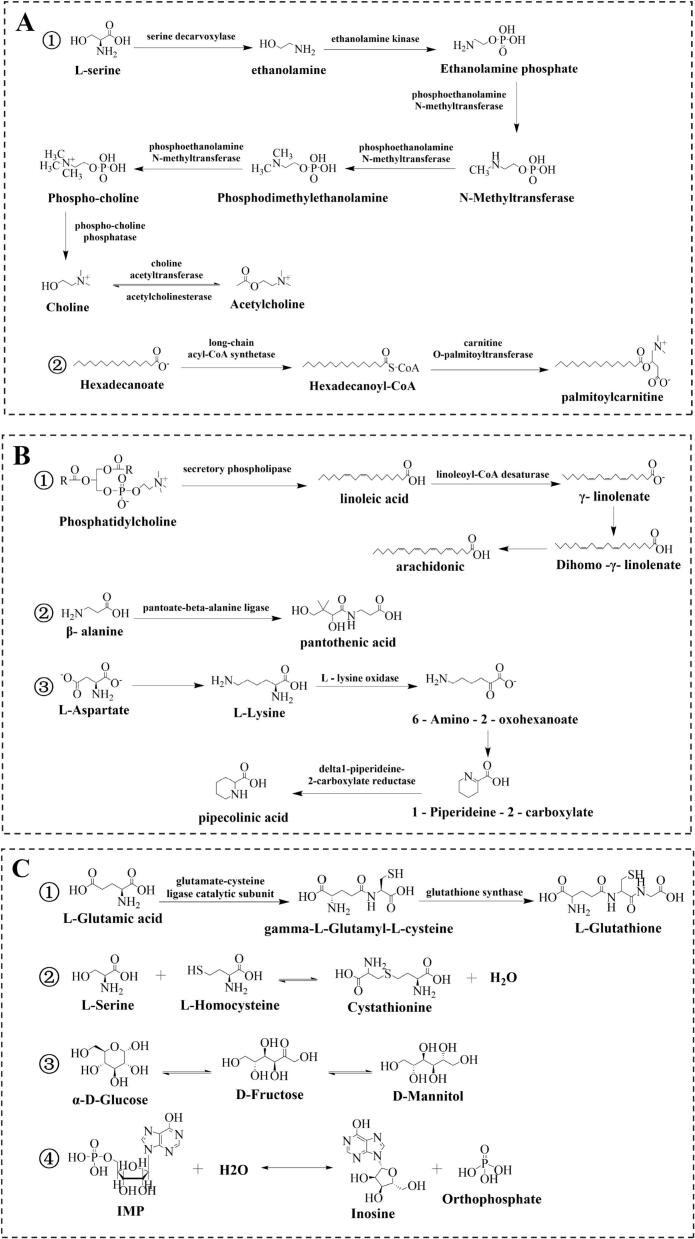
Metabolic pathway diagrams for key metabolites in four famous freshwater fish in China. Metabolic pathways of key lipids and analogues (A); Metabolic pathways of key organic acids (B); Metabolic pathways of other key metabolites (C).

According to the KEGG analysis, a total of 3 KEGG pathways involving key lipids had been identified (Table S4). Choline participated in the metabolism of serine and glycerophospholipids, and acetylcholine participated in the metabolism of glycerophospholipids. Besides, the metabolic pathways in Fig. 5A showed that choline and acetylcholine can be converted to each other under the action of enzymes, and serine was served as a precursor for choline and acetylcholine metabolism. The function of carnitine in reducing body fat accumulation lies in its ability to transport fatty acids to mitochondria so that β-oxidation and energy production can occur. Palmitoylcarnitine is a crucial player in fatty acid metabolism. As shown in Fig. 5C, palmitoylcarnitine content was observed as purple in AN (high content) while green (low content) in the other three fishes (green), Consequently consuming AN may be more beneficial for people with weight loss needs than the other three fishes. However, it has been reported that the content of palmitoylcarnitine increased after steaming (Li, Sun, et al., 2021). Moreover, further research is needed to explore how the processed forms influence the content of palmitoylcarnitine in these four fish.

##### 3.7.2.2 Organic acids

A total of 9 organic acids were identified as key metabolites through screening. KEGG analysis revealed the presence of 3 metabolic pathways associated with 4 key metabolites (Table S4). As shown in Fig. 5B, 2 of metabolic pathways were related to amino acids and 1 related to organic acids. Notably, arachidonic, oleic, and linoleic acids not only serve as essential fatty acids, but also act as important precursors for volatile compounds (Qiu et al., 2023). Arachidonic acid participates in the metabolism of linoleic acid, while both arachidonic acid and linoleic acid can be metabolized from phosphatidylcholine via a series of enzymes reactions. 1-octen-3-ol with mushroom flavor is an important volatile compound found in fish (Jiang et al., 2017). Ding et al. (2020) demonstrated a highly significant positive linkage linking the content of 1-octen-3-ol to that of linoleic acid. MP had the highest content of linoleic acid among the four fish species (Fig. 5C), suggesting MP has the highest 1-octen-3-ol content, consequently, it will exhibit a stronger mushroom flavor compared to the other three fish species. Additionally, β-alanine generates pantothenic acid by pantoate–beta-alanine ligase. l-aspartate can be converted to l-lysine, and l-Lysine is a metabolite precursor of pipecolinic acid. In conclusion, amino acids are crucial flavor components in these four fish, serving as both key flavor agents and essential flavor precursors.

##### 3.7.2.3 Other key metabolites

The critical metabolites we identified included inosine-5′-monophosphate (IMP), a nucleotide that significantly influences the freshness and flavor of fish. It should be noted that as the level of IMP in the muscle increases, the flavor of this meat product becomes stronger (Liu, Wen, et al., 2024). As shown in the heatmap (Fig. 4C), the red coloration observed for IMP in all the four fish species indicated that high levels of this metabolite. Comparatively, CI had a darker red color, indicating that its meat freshness may be more pronounced compared to the other three fish species. It is important to highlight that the IMP content identified by LC-MS differs from the IMP levels previously detected in the nucleotides and their derivatives of four famous freshwater fish in China. This discrepancy may be attributed to variations in the assay environment and conditions. In addition, inosine was also detected as a significant metabolite, which can be generated by IMP metabolism (Fig. 5C). It has been shown that an accumulation of inosine in chicken breast meat resulted in an unpleasantly bitter taste (Liu et al., 2021).

Both Cystathionine and L-Glutathione are amino acids derivatives. Cystathionine is a metabolic intermediate for sulfur-containing amino acids, which can be produced through the action of l-Serine and L-Homocysteine. On the other hand, L-Glutathione is an antioxidant (Setyabrata et al., 2021), and it is synthesized through a series of enzymatic reactions from L-Glutamic acid, involving enzymes such as glutamate—cysteine ligase catalytic subunit and glutathione synthase. The content of L-Glutathione in CI and HM is higher than that in MP and AN, suggesting that MP and AN are more susceptible to oxidation and even rancidity when compared to CI and HM. However, it is important to note that this comparison is relative.

In summary, this research mainly analyzes the differences in taste profiles among the four famous fish species, which may be attributed to the content of flavor amino acids, nucleotides and their derivatives, as well as to free amino acid metabolism, lipid metabolism, and organic acid metabolic pathways. Moreover, the interaction effects between the key differential substances and their impact on the flavor quality of the four major fish species still require further study. Actually, the formation of aquatic product flavor is influenced by many factors, mainly including varietal genetics, growth environment, and source of nutrients,. For instance, MP prefers the bottom of the water and feeds on snails, clams, etc. CI mostly inhabits the middle and lower layers of the water and loves to eat aquatic plants; HM is active in the upper layer of the water and feeds on planktonic plants; AN is in the middle-upper layer of the water and mainly feeds on floating animals (Song & Cheng, 2023). Besides, climate, geographical conditions, salinity, temperature, feed sources, storage and transportation conditions, etc., all have an impact on the quality and flavor characteristics of aquatic products. When conducting market sampling, it is difficult to attribute changes in flavor to individual factors. To investigate the impact of a single variable on flavor, experiments must be performed under controlled conditions. In a word, this study initially identifies the chemical profiles of four renowned fish species with distinct flavor types and explores the material basis underlying their flavor formation. The aim is to conduct an in-depth analysis of the fundamental taste characteristics of these major fish species, providing a scientific foundation for the rational development of related products and advancing the deep processing and quality enhancement of aquatic raw materials.

## 4 Conclusion

In this study, we explored the structural characteristics, taste attributes, and non-volatile metabolites of the four famous freshwater fish in China using techniques such as TPA, SEM, *E*-tongue, LC-MS, free amino acid analysis, and the determination of nucleotides and their derivatives. The results indicated that although the microstructures of the four species were similar, MP and AN exhibited superior tenderness compared to CI and HM. Notably, MP had a significantly higher content of total free amino acids (FAAs) than the other three fish species (*p* < 0.05). The analysis of nucleotides and their derivatives suggested that HM and MP likely demonstrated greater freshness, while CI appeared to be more prone to spoilage after slaughter. E-tongue analysis showed that AN and MP had similar flavors profiles while CI and MP potentially possessed more pronounced taste flavors than AN and HM. The difference in flavor of the four fish species is primarily originated from free amino acid metabolism, lipid metabolism, and organic acid metabolic pathways. PLS-R analysis revealed that umami and other sensory attributes emerge from synergistic interactions among multiple compounds rather than the effect of a single taste-producing substance. In conclusion, the analysis of taste and flavor variations among four famous freshwater fish in China can provide valuable insights for consumers in their selection of preferred fish. Moreover, these findings serve as a vital cornerstone for improving the texture and flavor of these carps, which may contribute to the progress of the food industry.

## CRediT authorship contribution statement

**Lili Chen:** Writing – review & editing, Writing – original draft, Supervision, Software, Resources, Investigation, Funding acquisition, Formal analysis, Conceptualization. **Chaochao Wang:** Software, Methodology, Investigation, Formal analysis, Data curation. **Yu Liu:** Writing – original draft, Visualization, Software, Methodology, Investigation, Formal analysis, Data curation, Conceptualization. **Yongcheng Wang:** Software, Methodology, Investigation, Formal analysis, Conceptualization. **Chunqing Bai:** Supervision, Resources, Project administration. **Yong Jiang:** Supervision, Software, Resources, Project administration. **Meilan Yuan:** Supervision, Software, Resources, Project administration. **Li Zhao:** Writing – review & editing, Visualization, Resources, Project administration, Funding acquisition, Conceptualization. **Renjie Wang:** Writing – review & editing, Supervision, Software, Resources, Project administration, Funding acquisition, Formal analysis.

## Declaration of competing interest

The authors declare that they have no known competing financial interests or personal relationships that could have appeared to influence the work reported in this paper.

## Data Availability

Data will be made available on request.

## Appendix A Supplementary data

Supplementary material. Image 1
