# Supplementary material for: Comparative analysis of the non-volatile metabolites and taste profiles of the four famous freshwater fish raw materials in China
Source: Food Chem X. 2026 Jan 30;34:103623. doi: 10.1016/j.fochx.2026.103623 (PMC12906139; doi:10.1016/j.fochx.2026.103623)
Supplement: Supplementary file 1 — Supplementary material. [file mmc1.docx]

**Supplementary materials**

| **ESI+** | **Validation** | **ESI-** | **Validation** |
| --- | --- | --- | --- |
| 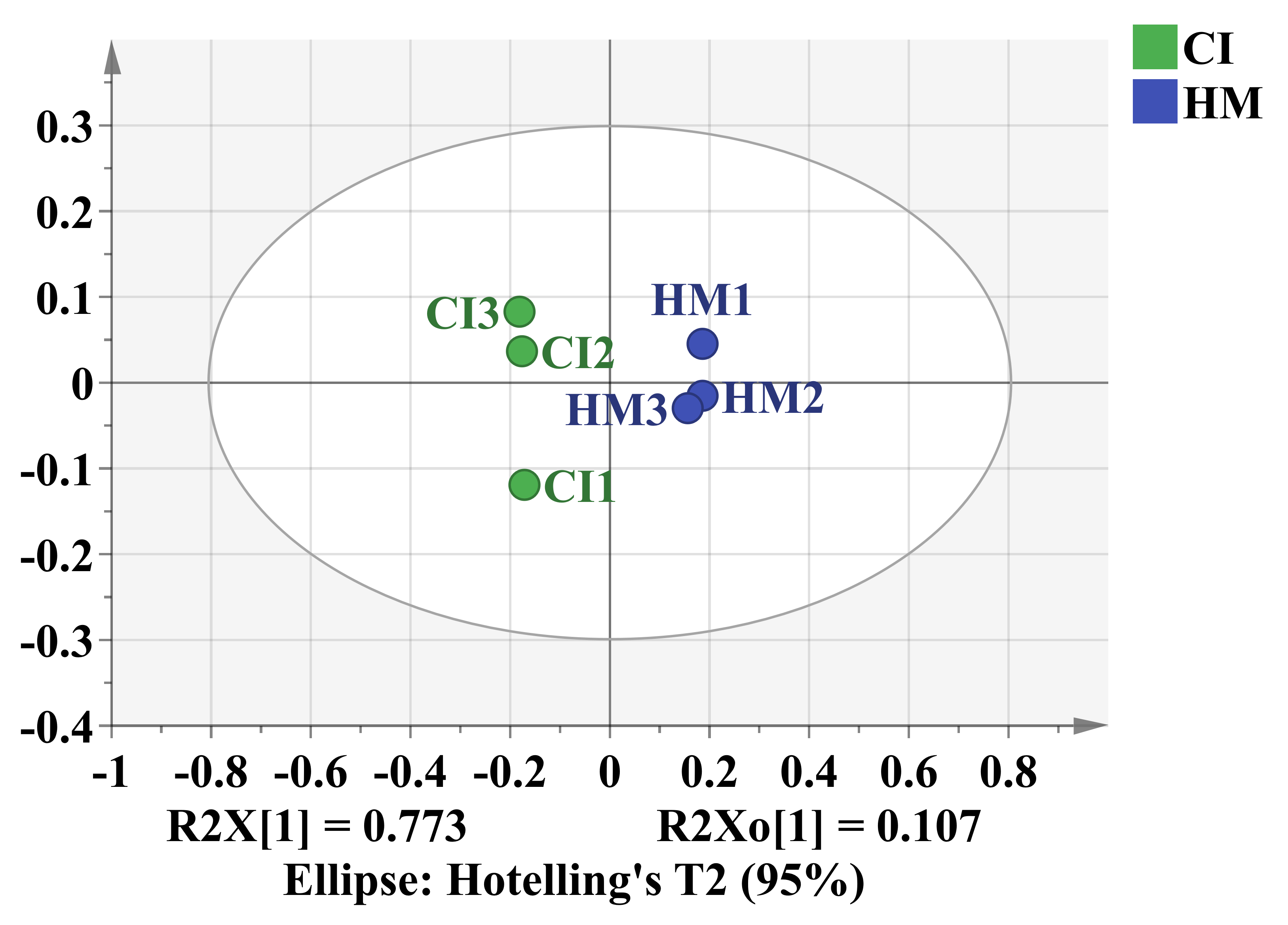 | 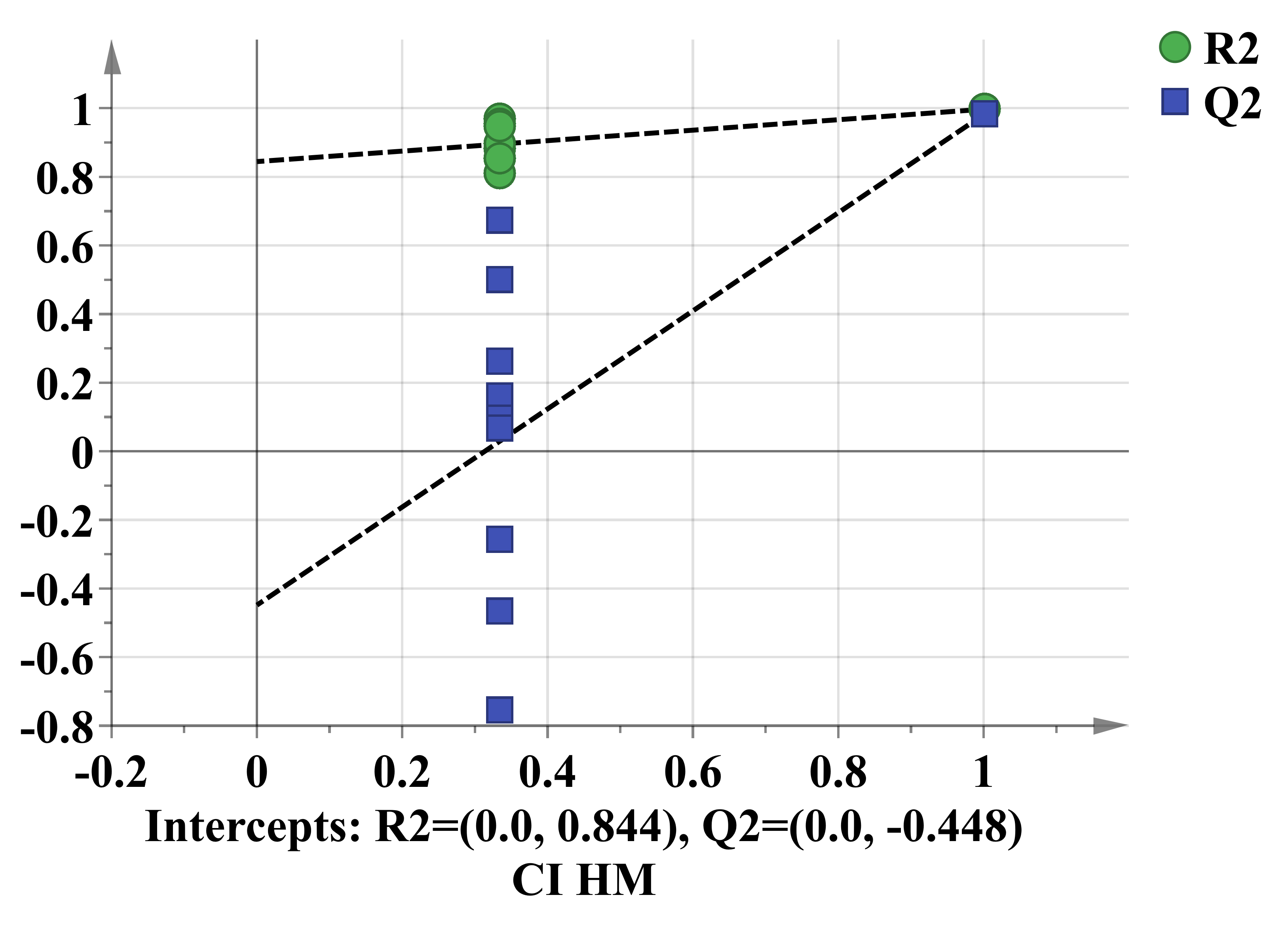 | 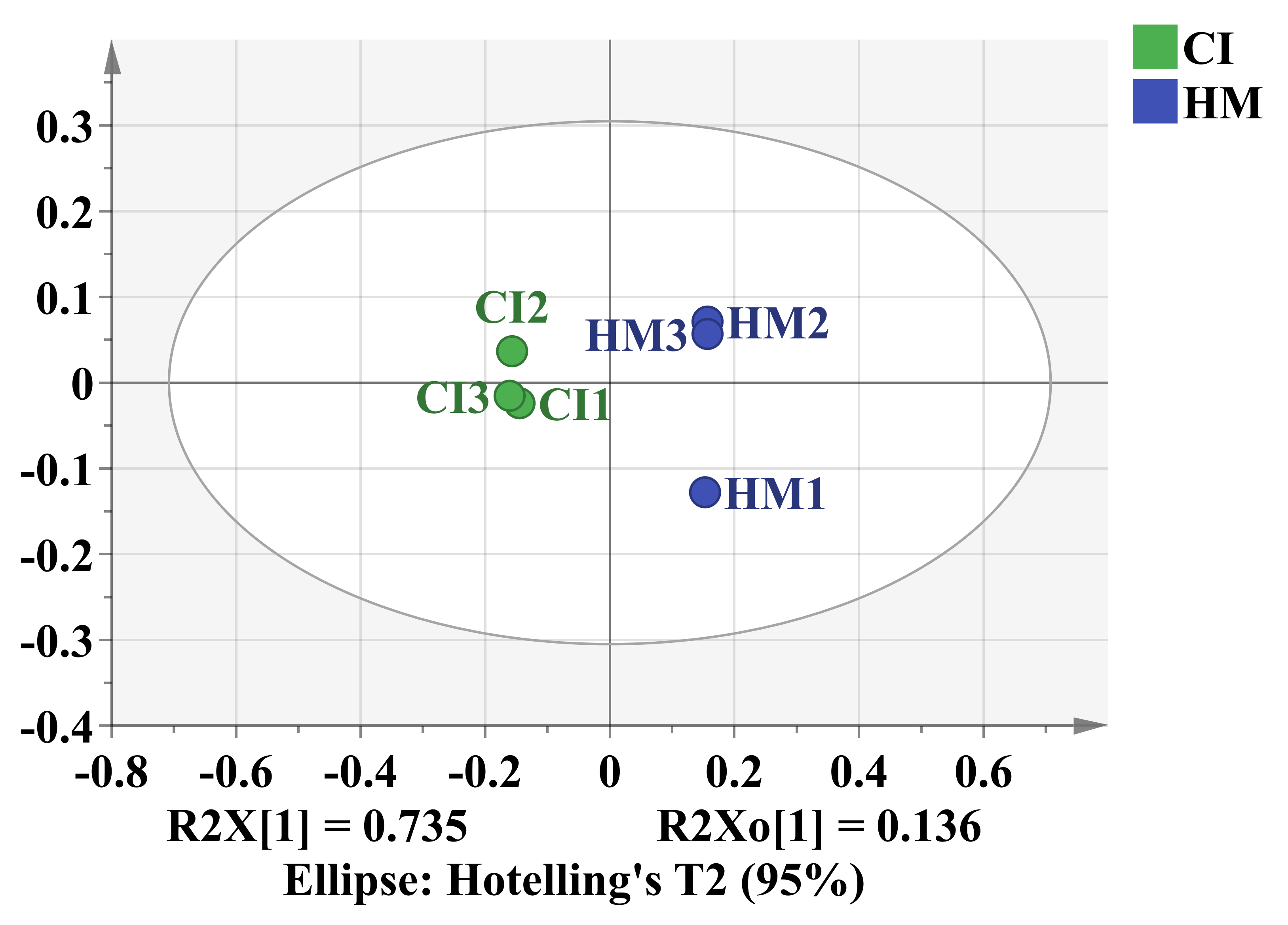 | 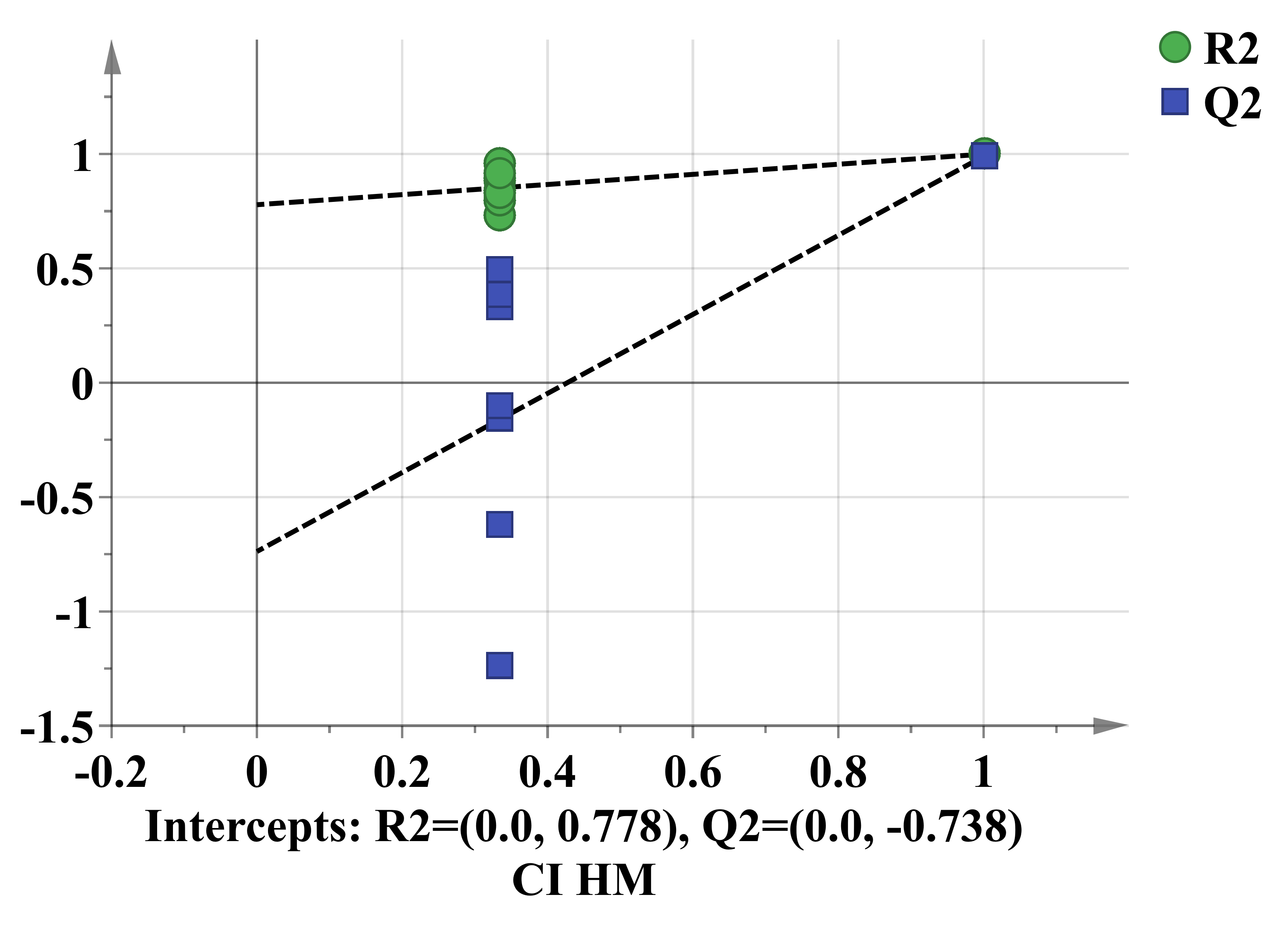 |
| 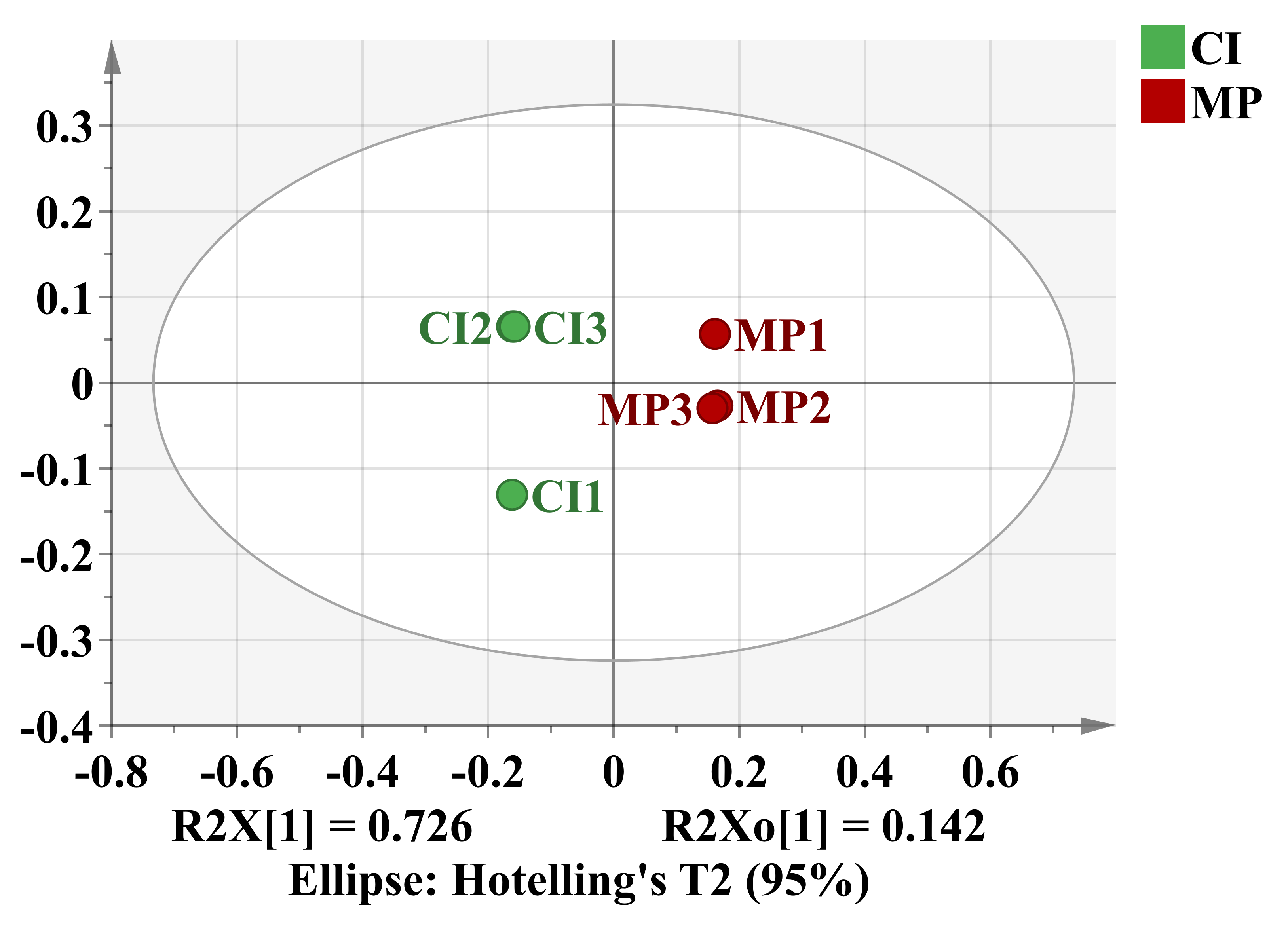 | 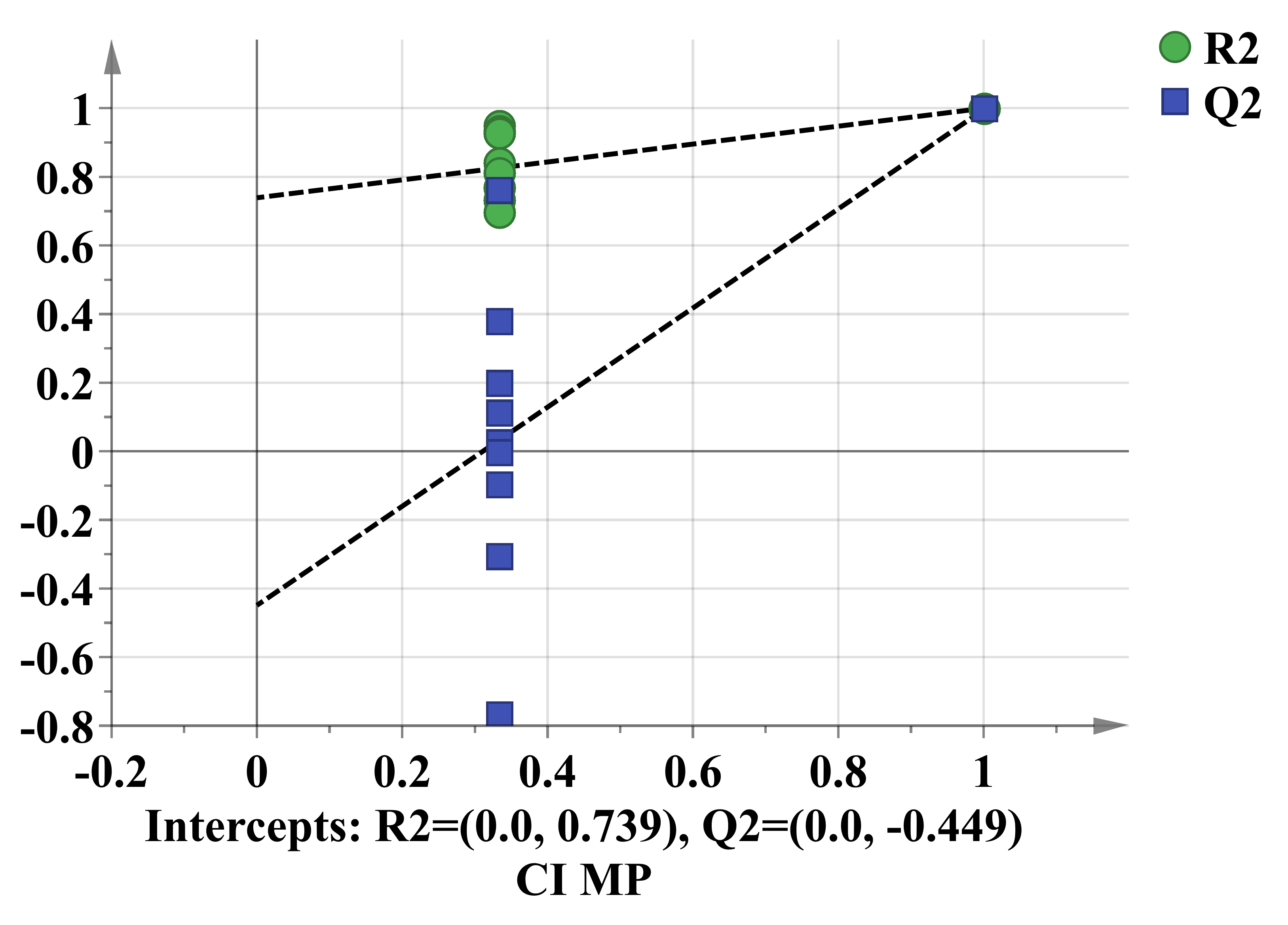 | 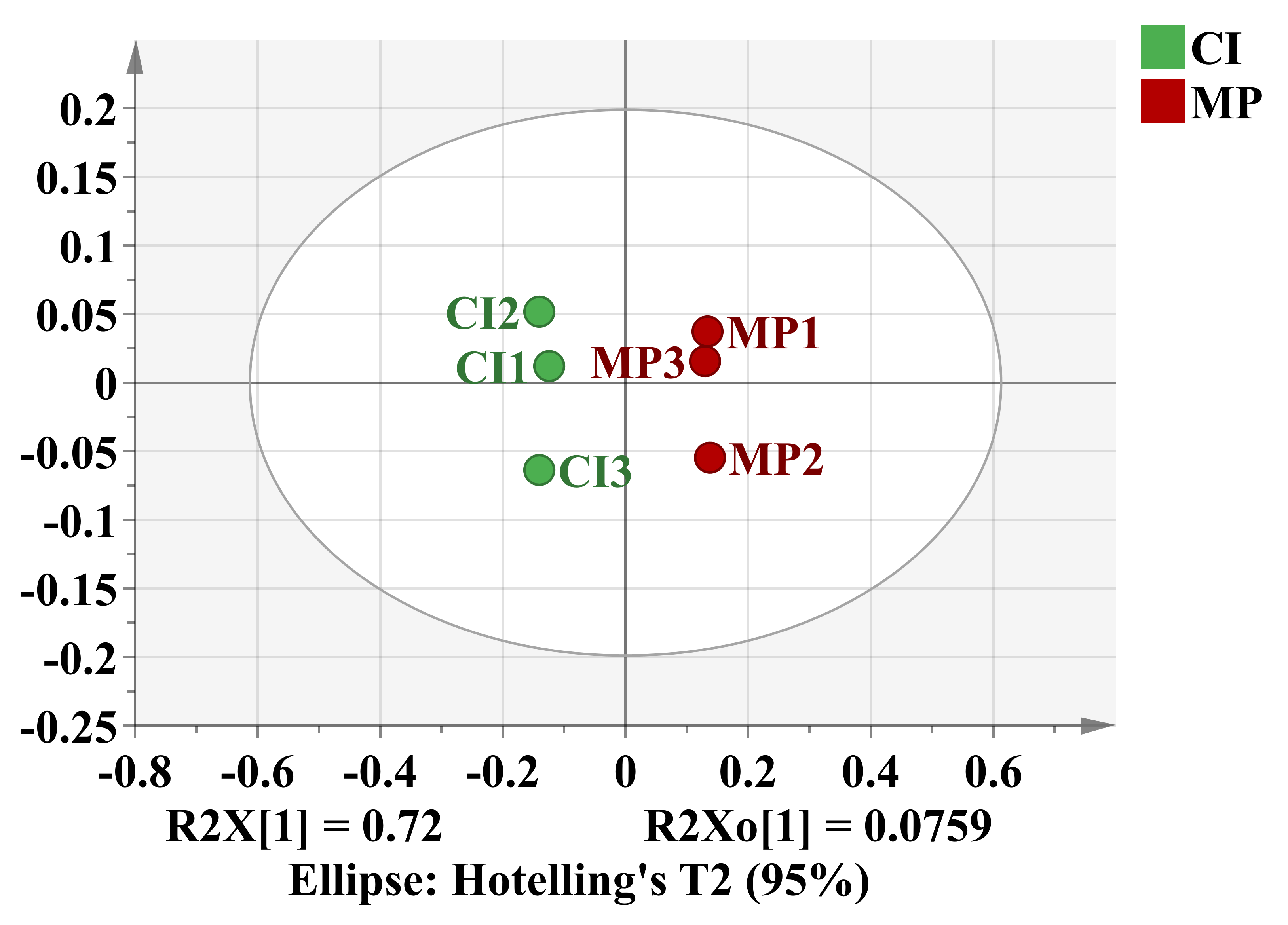 | 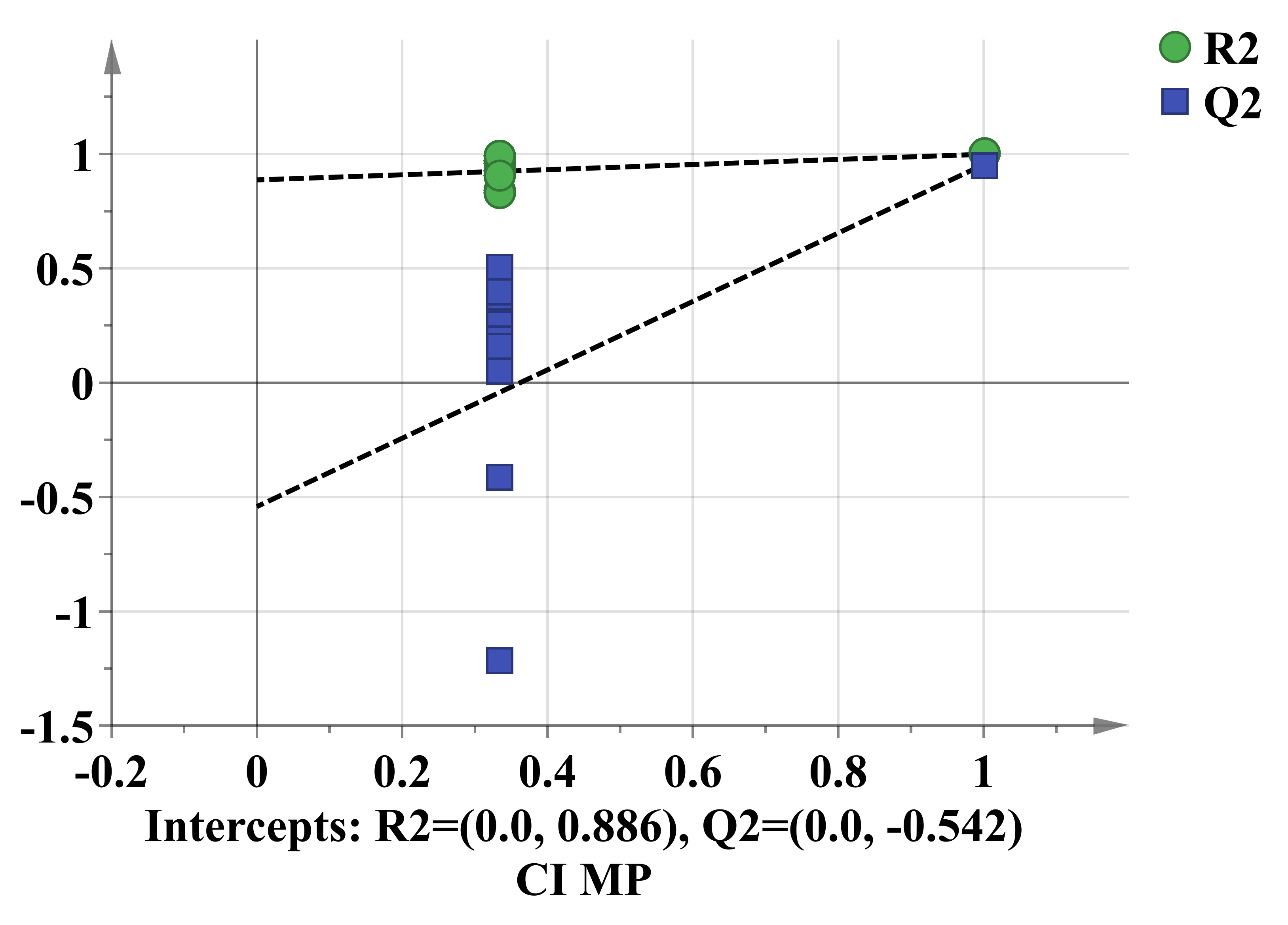 |
| 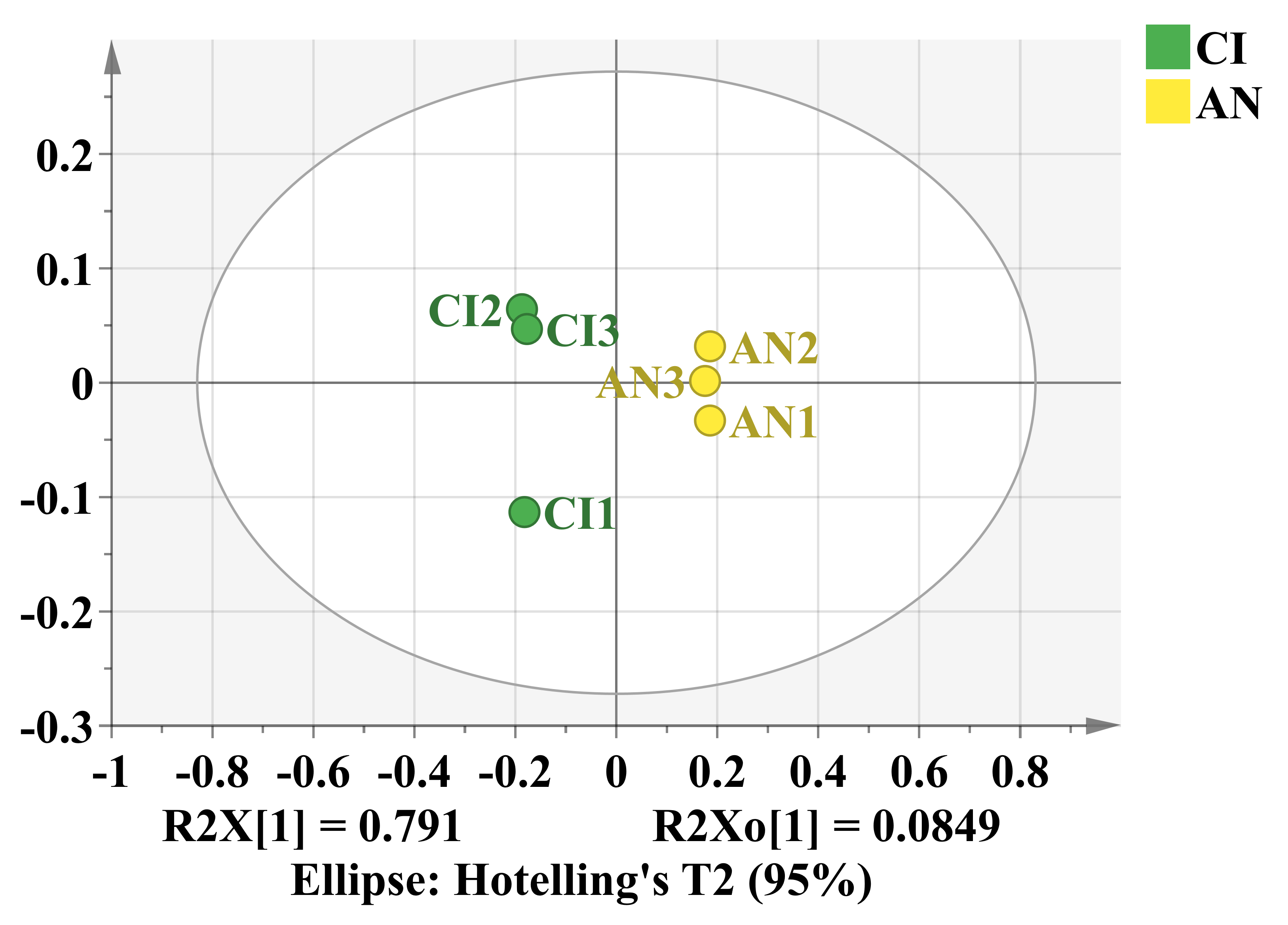 | 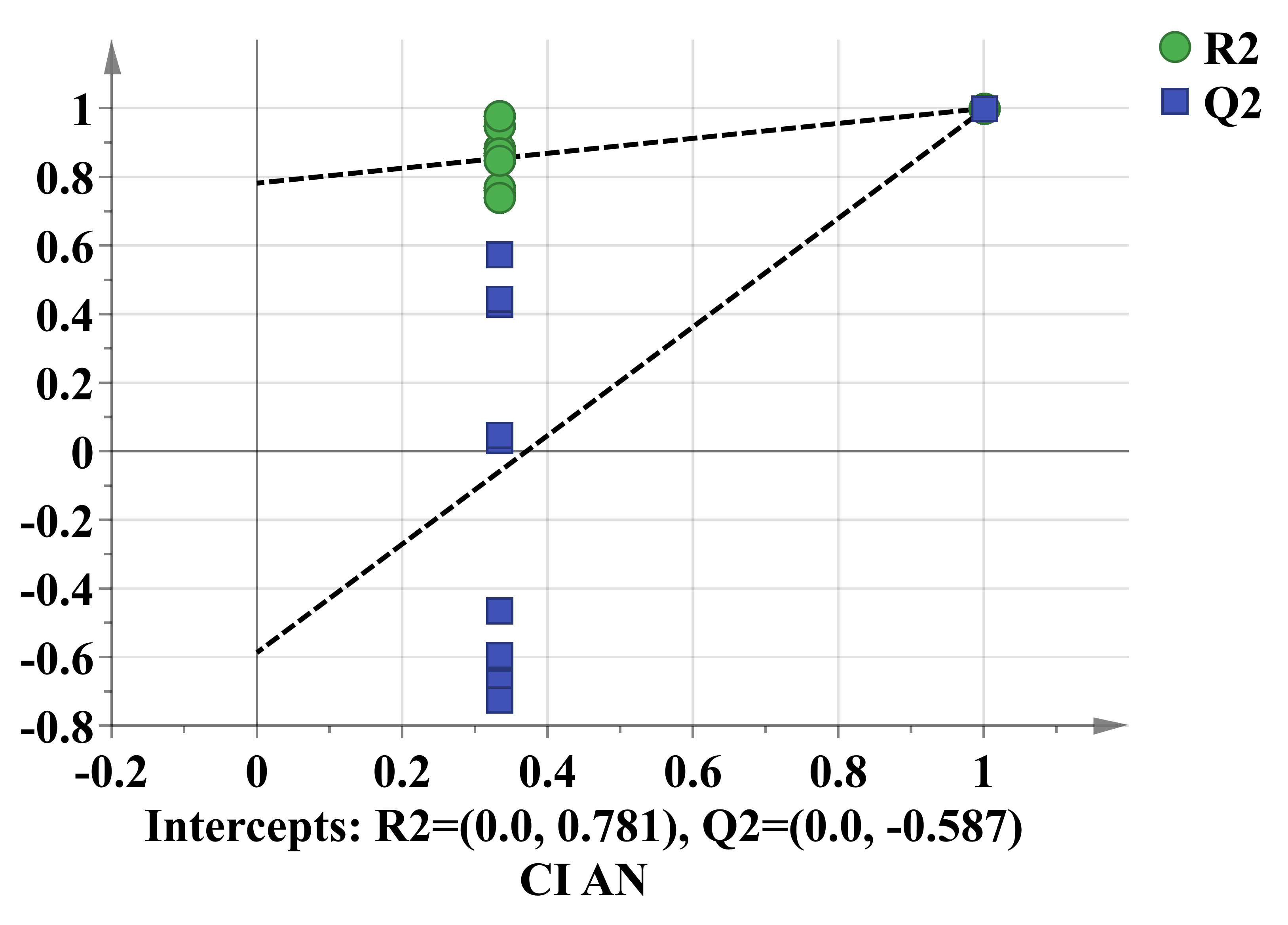 | 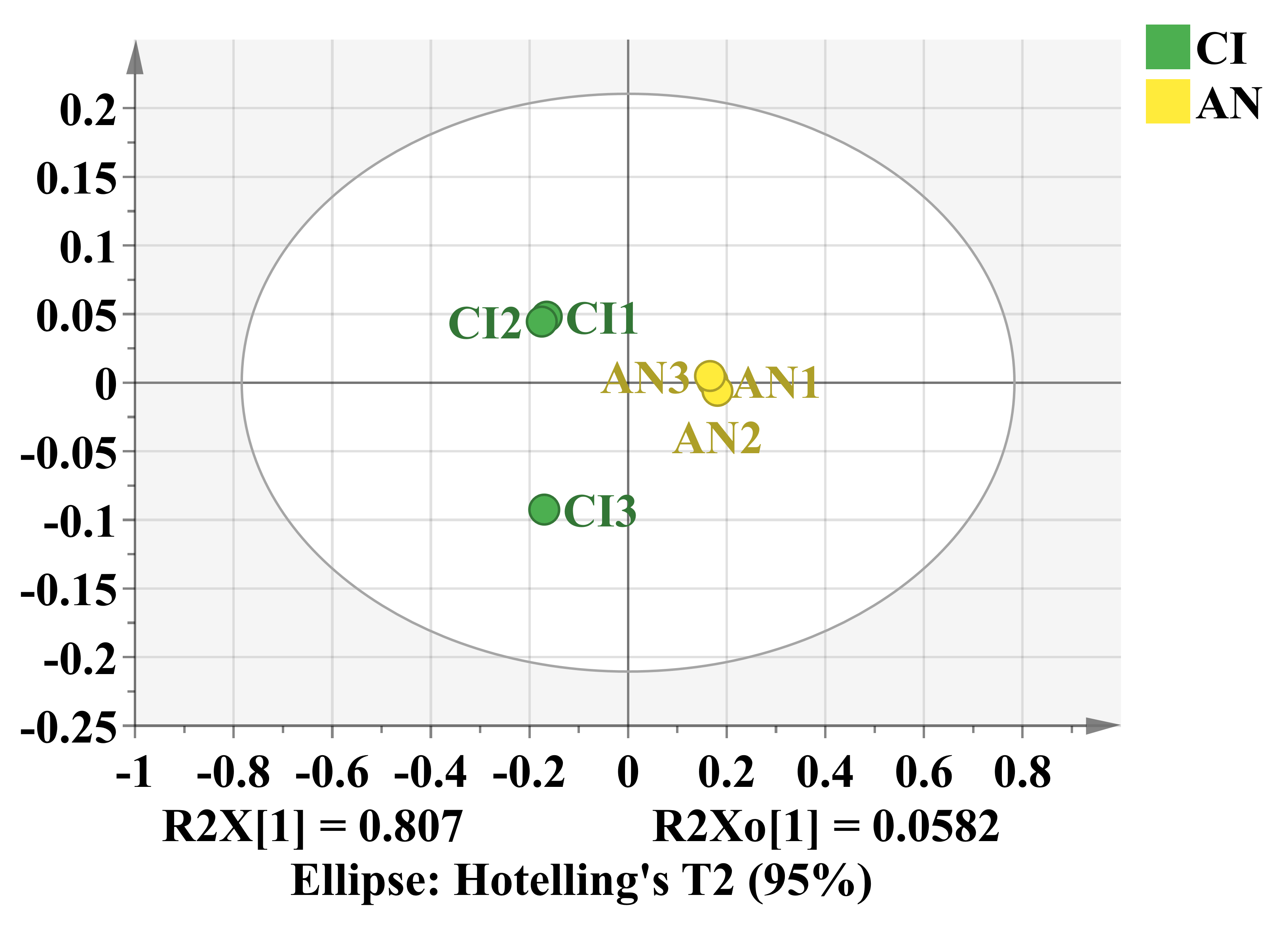 | 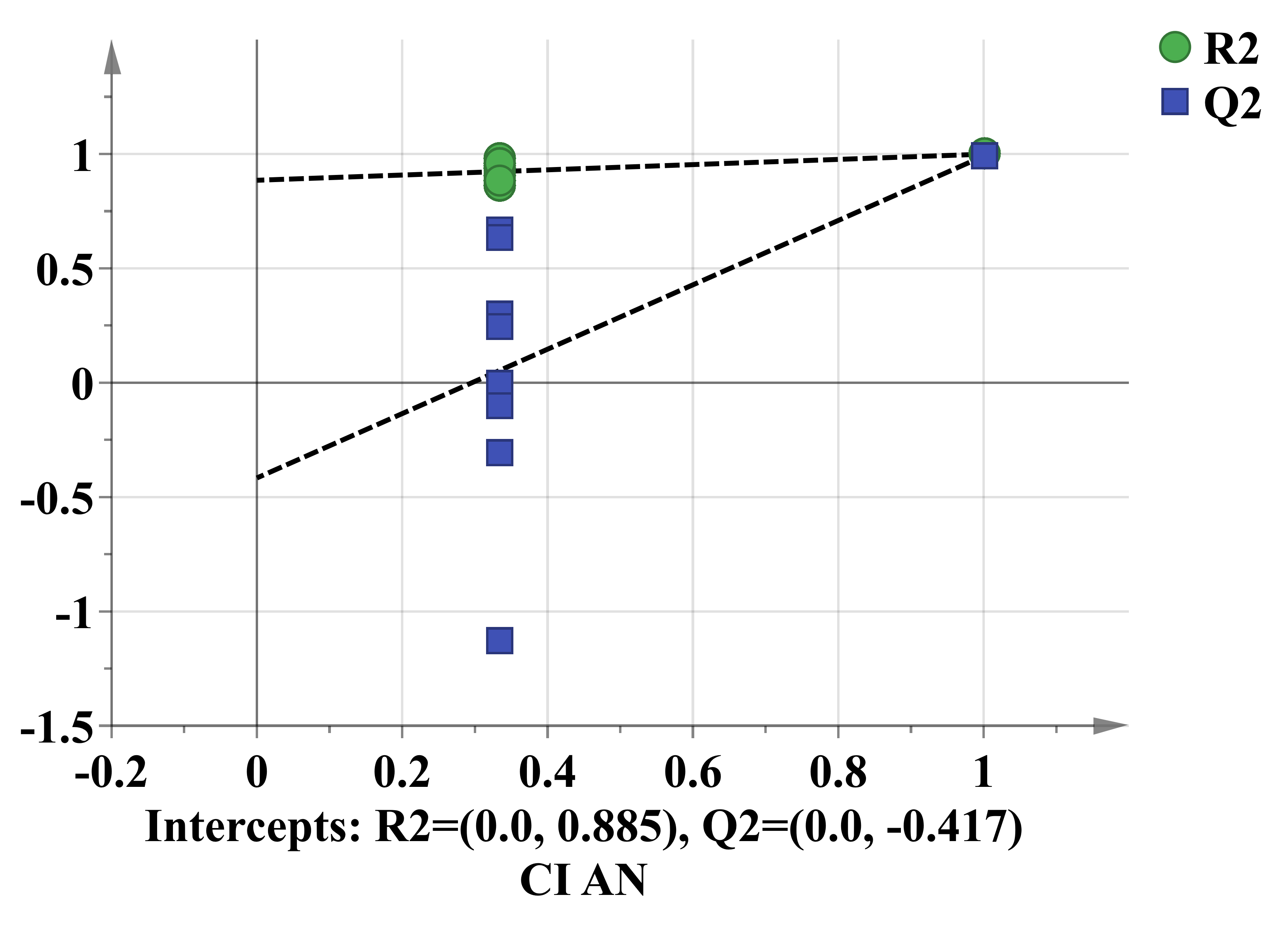 |
| 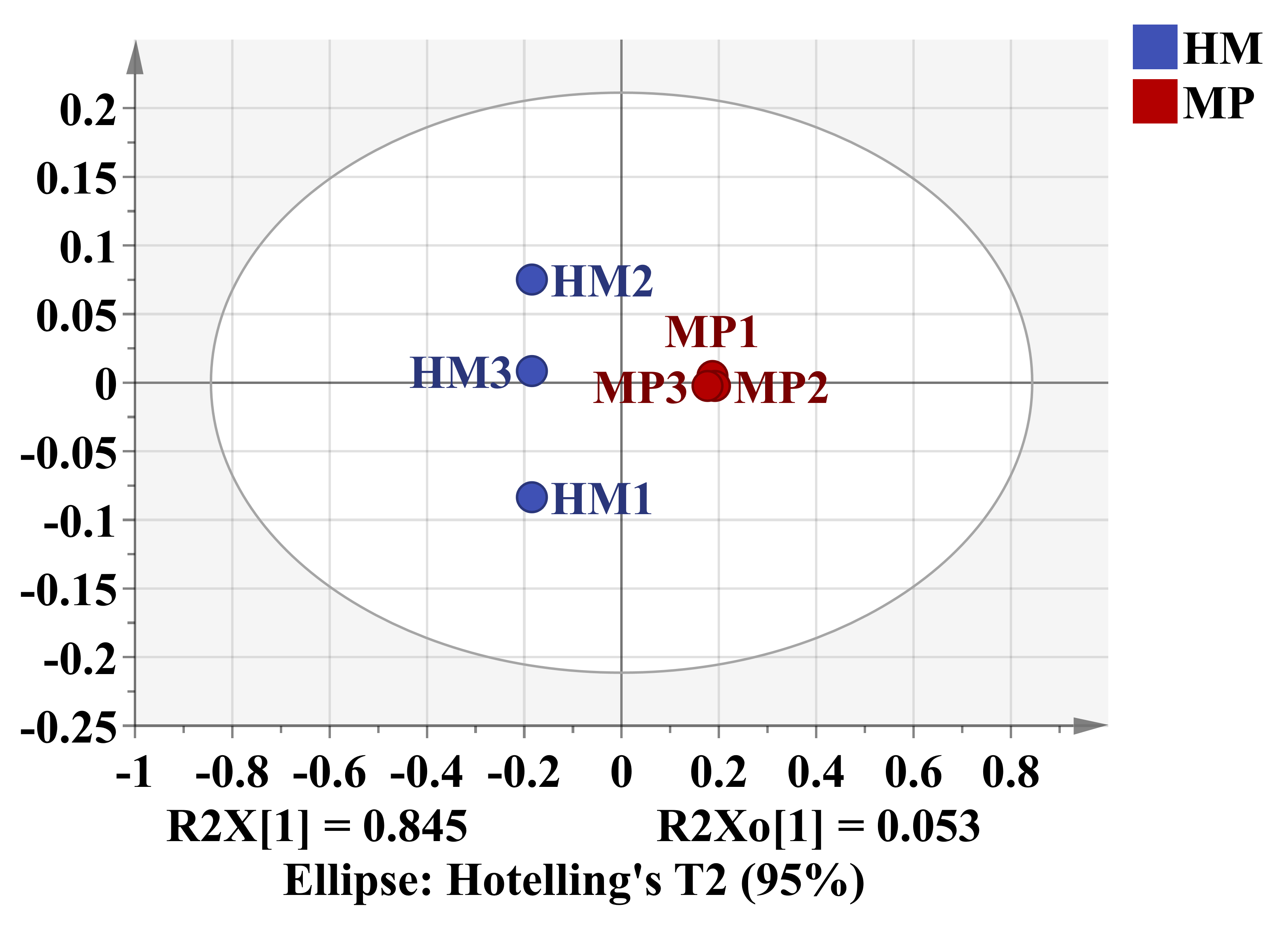 | 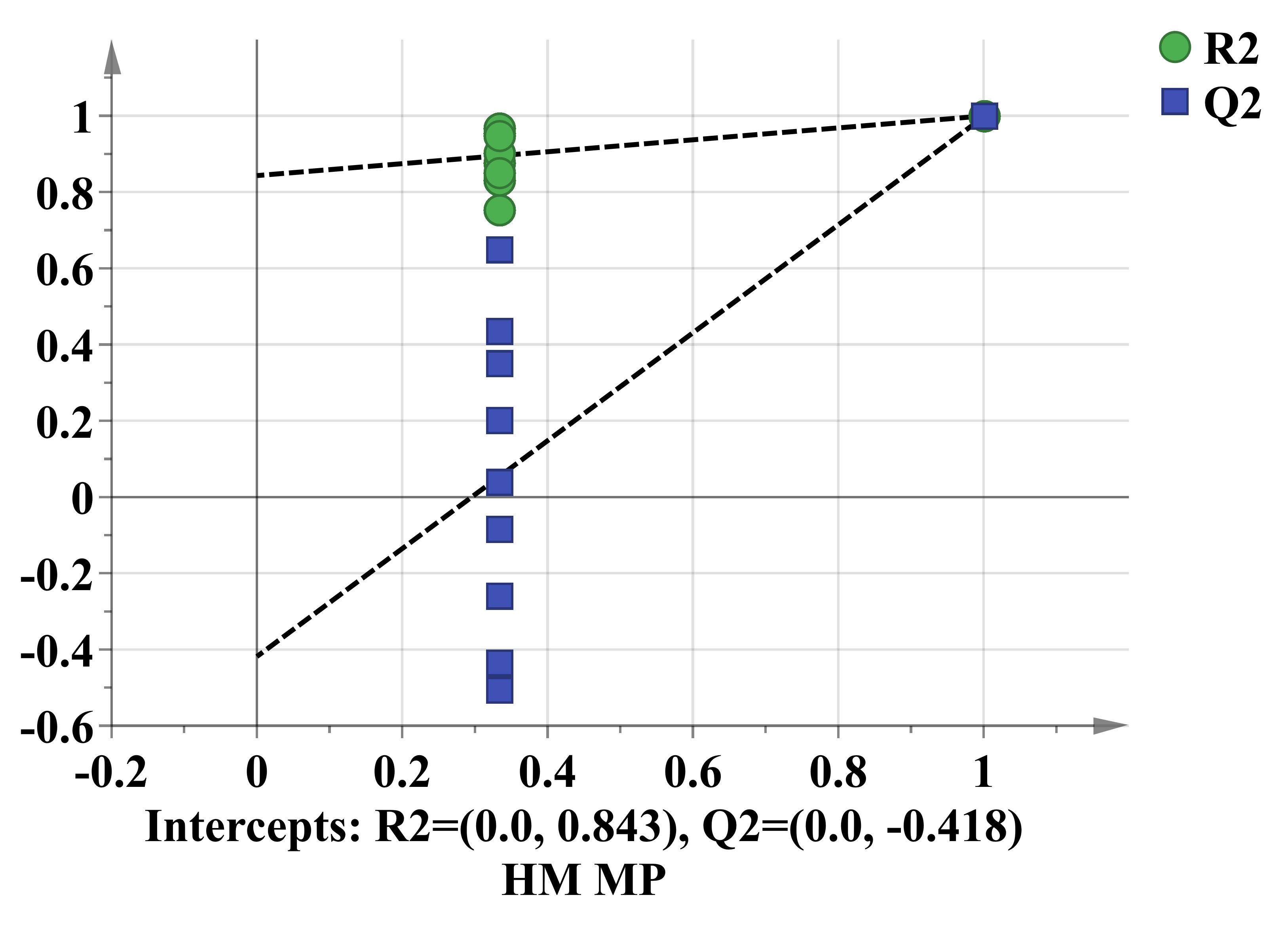 | 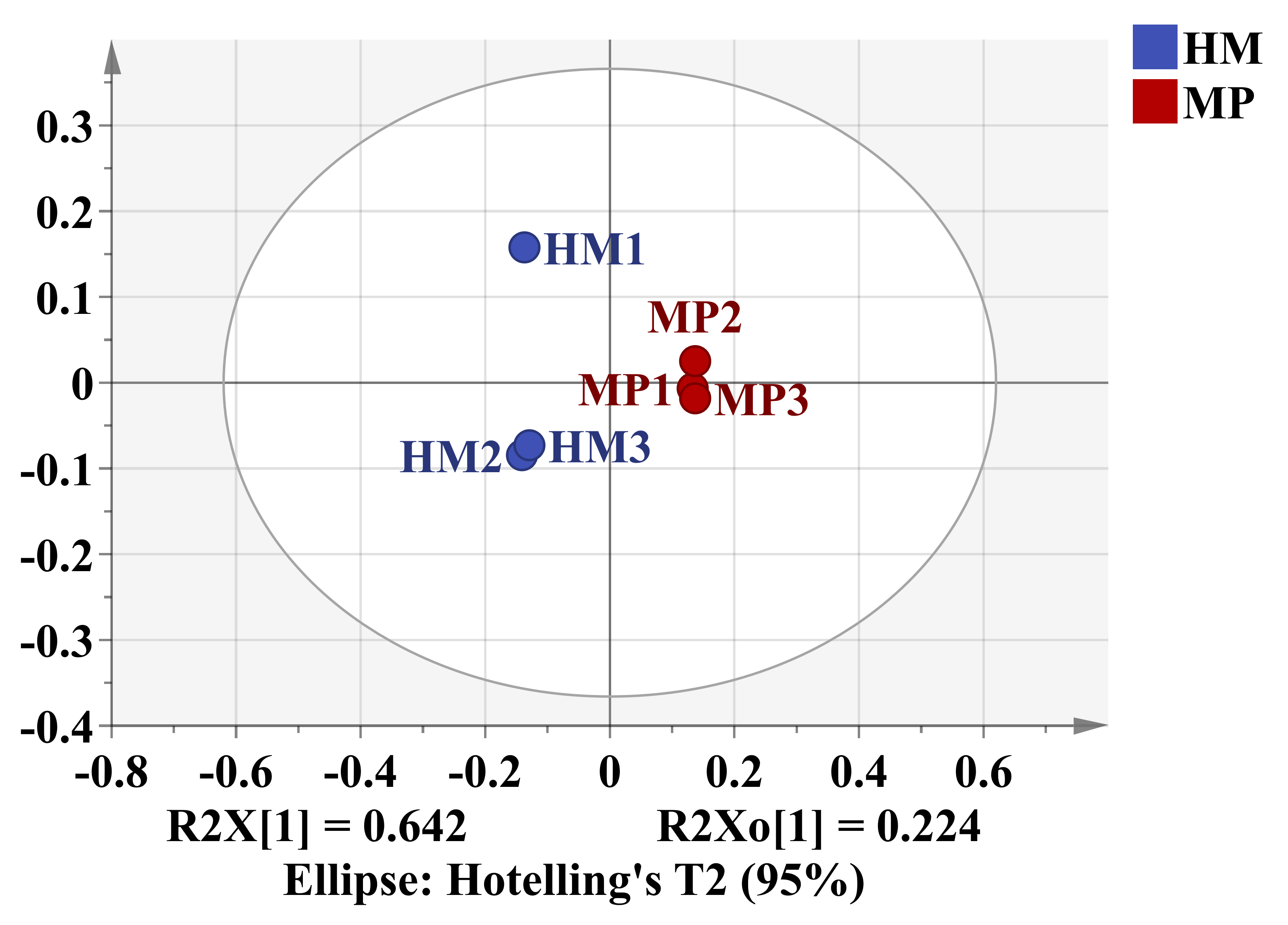 | 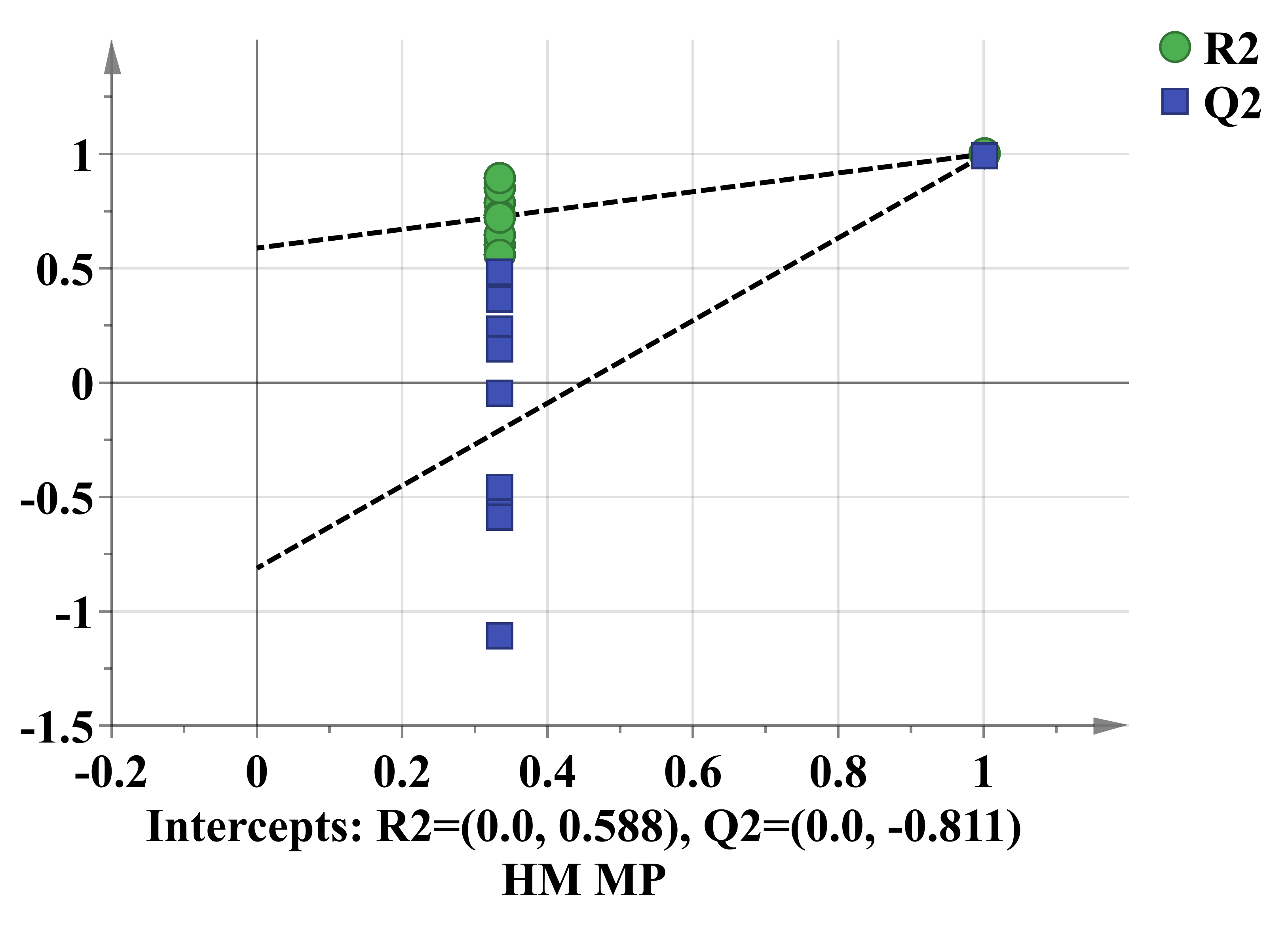 |
| 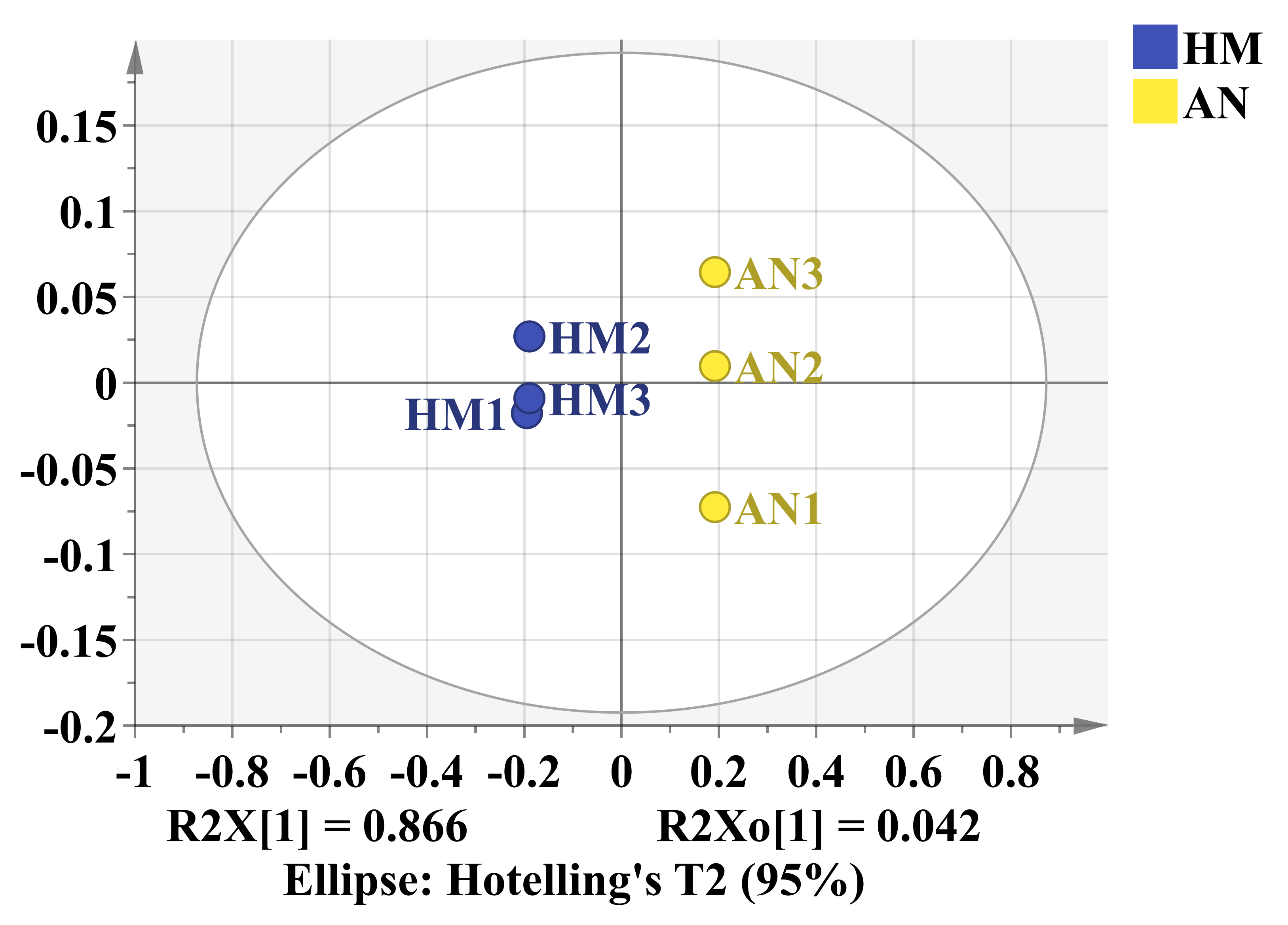 | 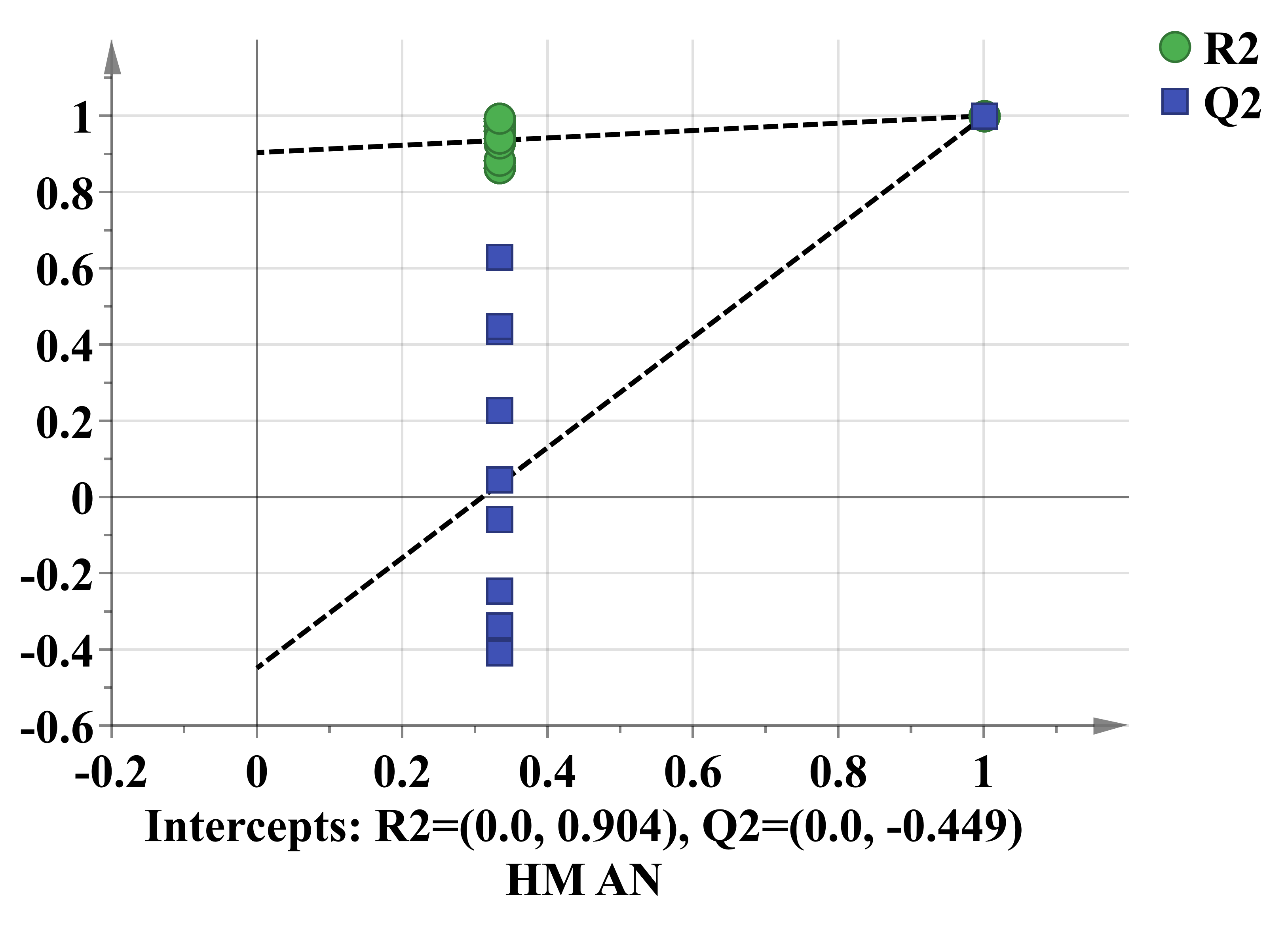 | 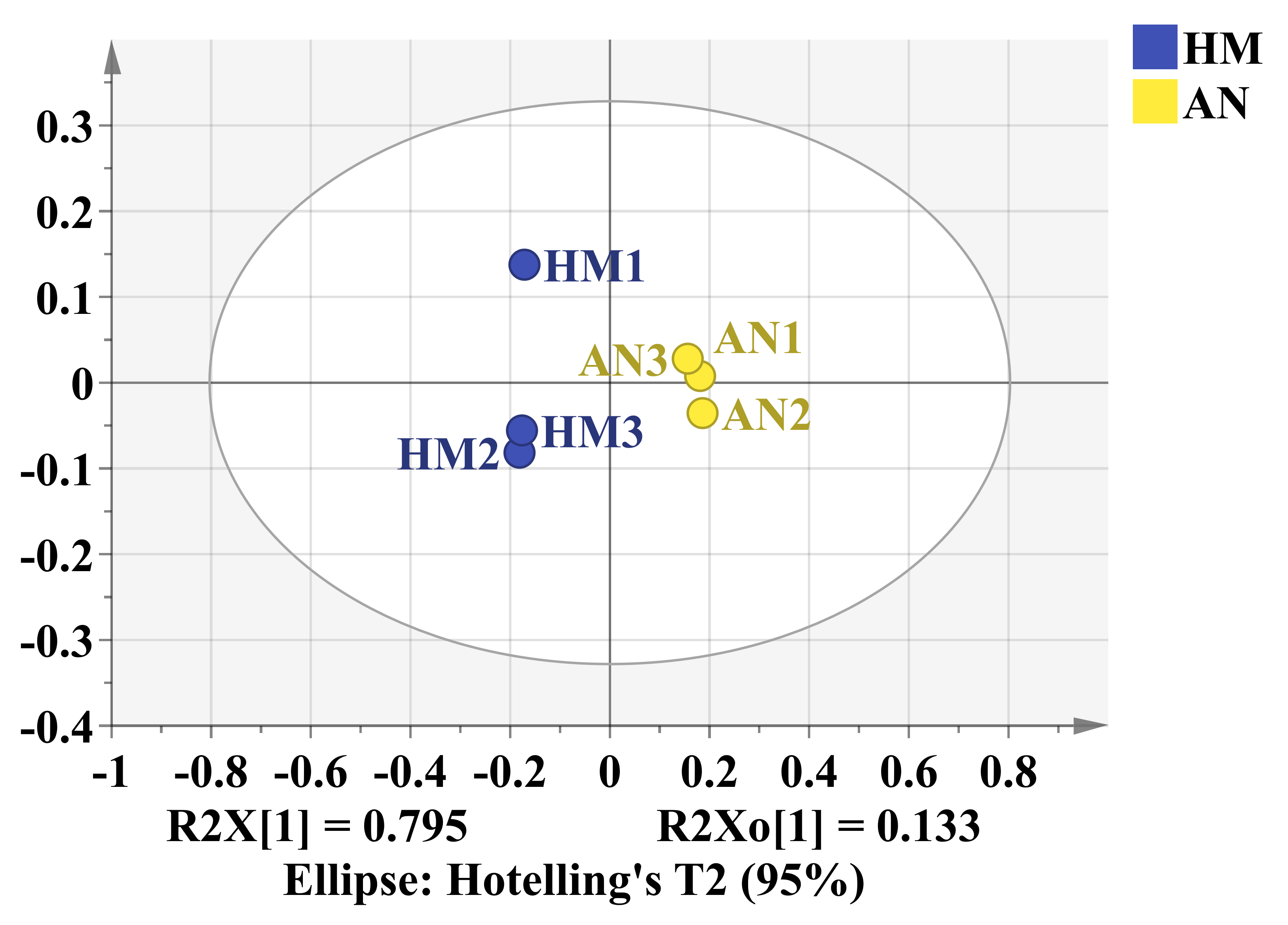 | 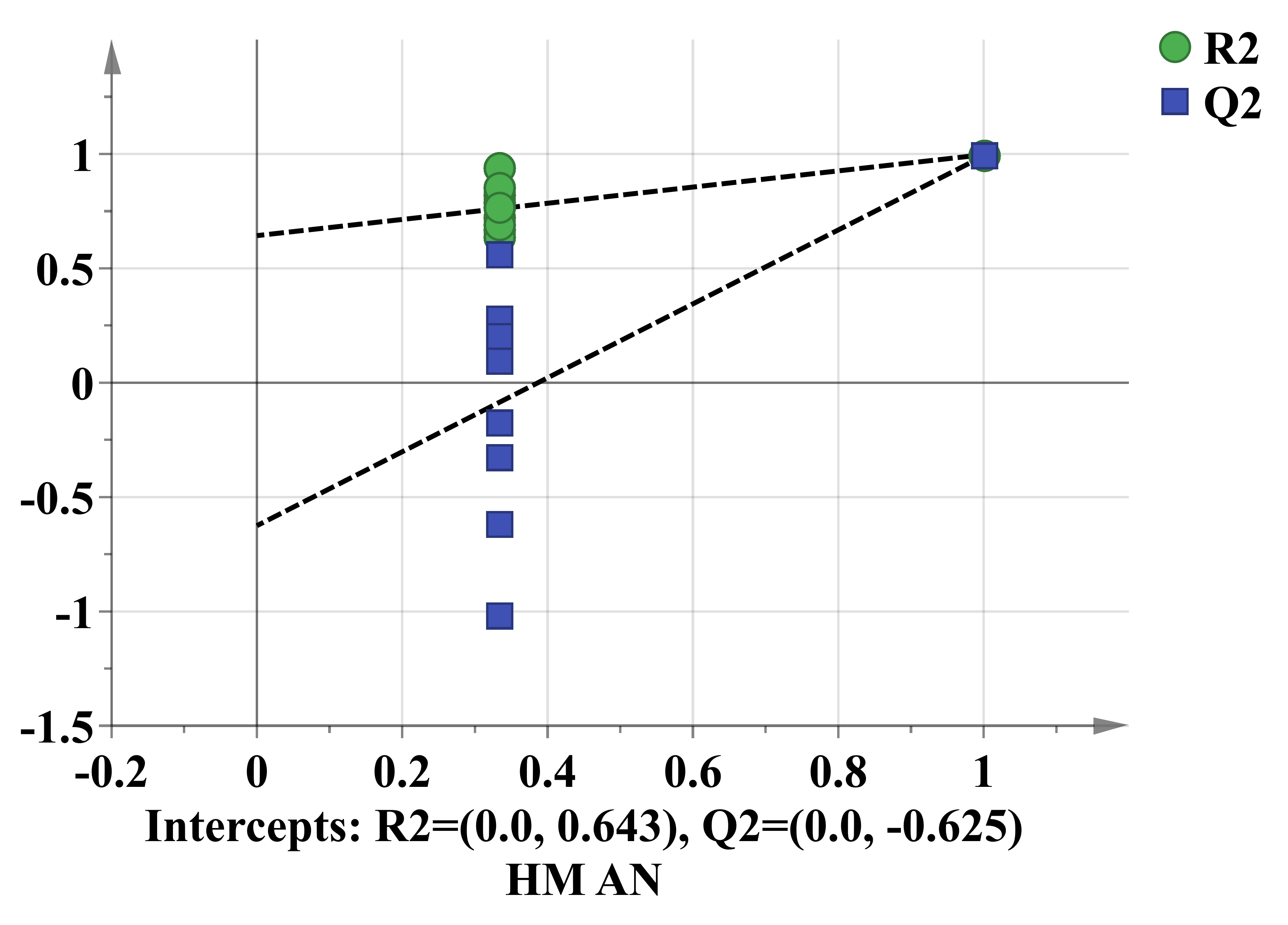 |
| 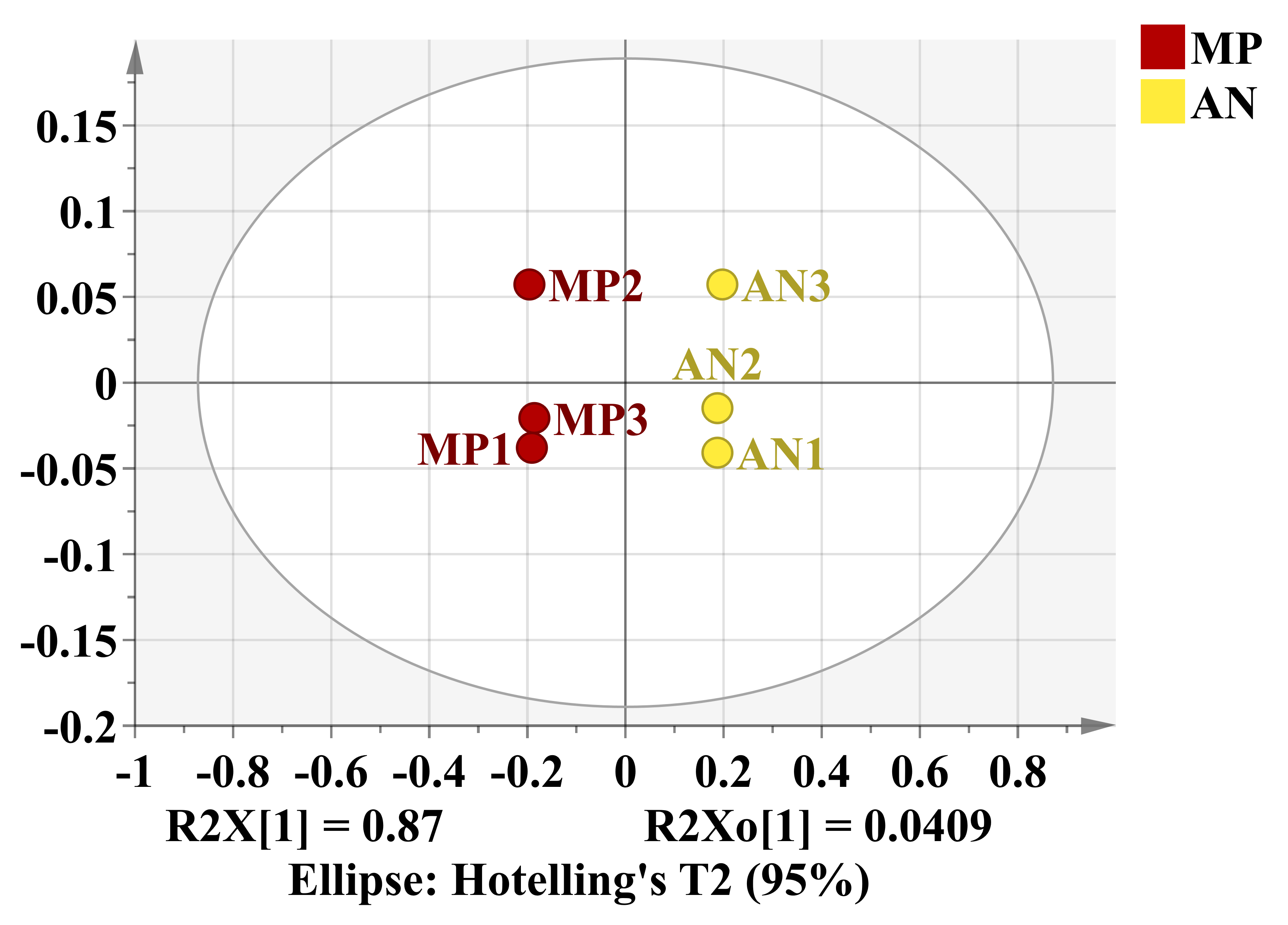 | 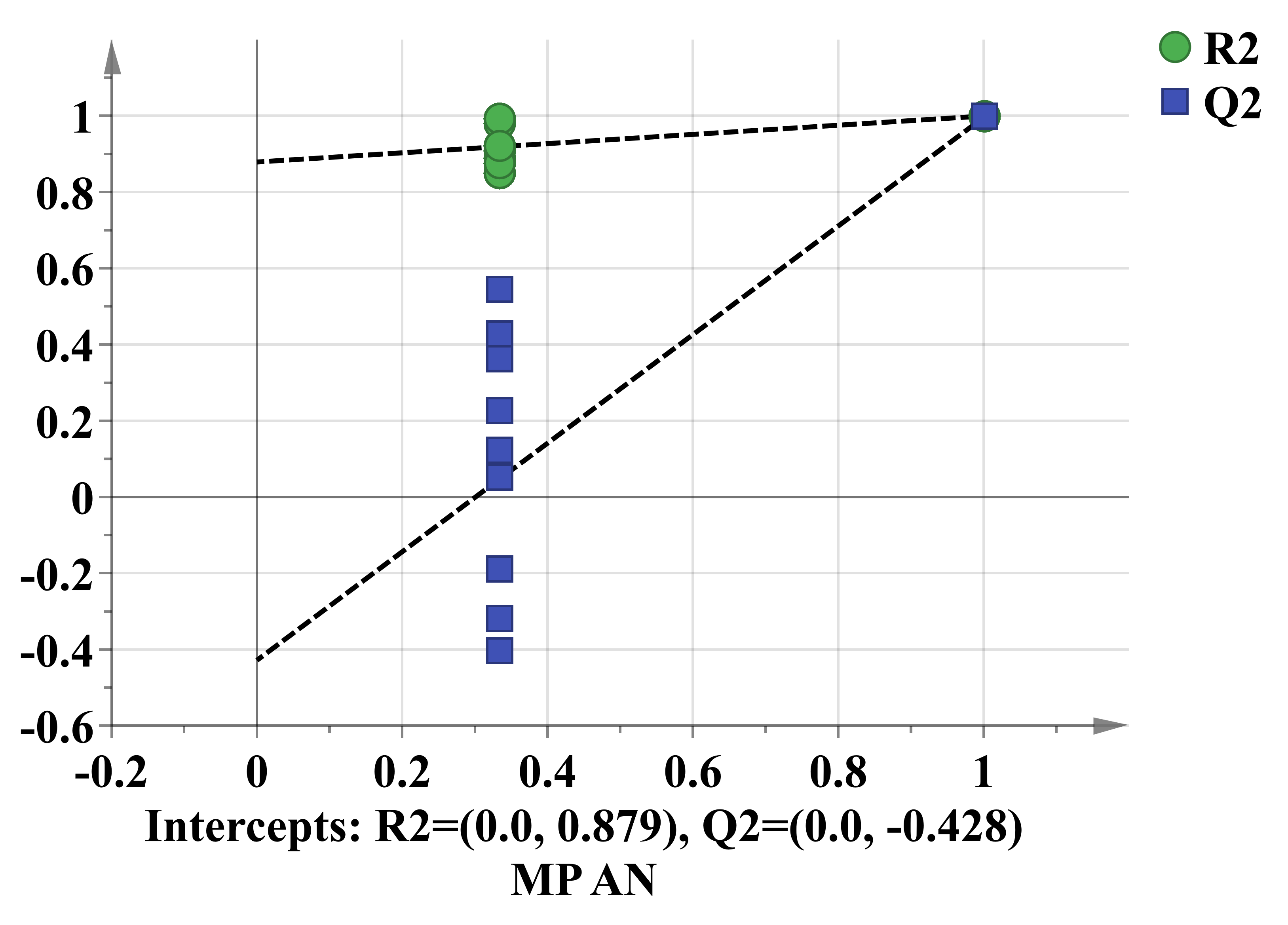 | 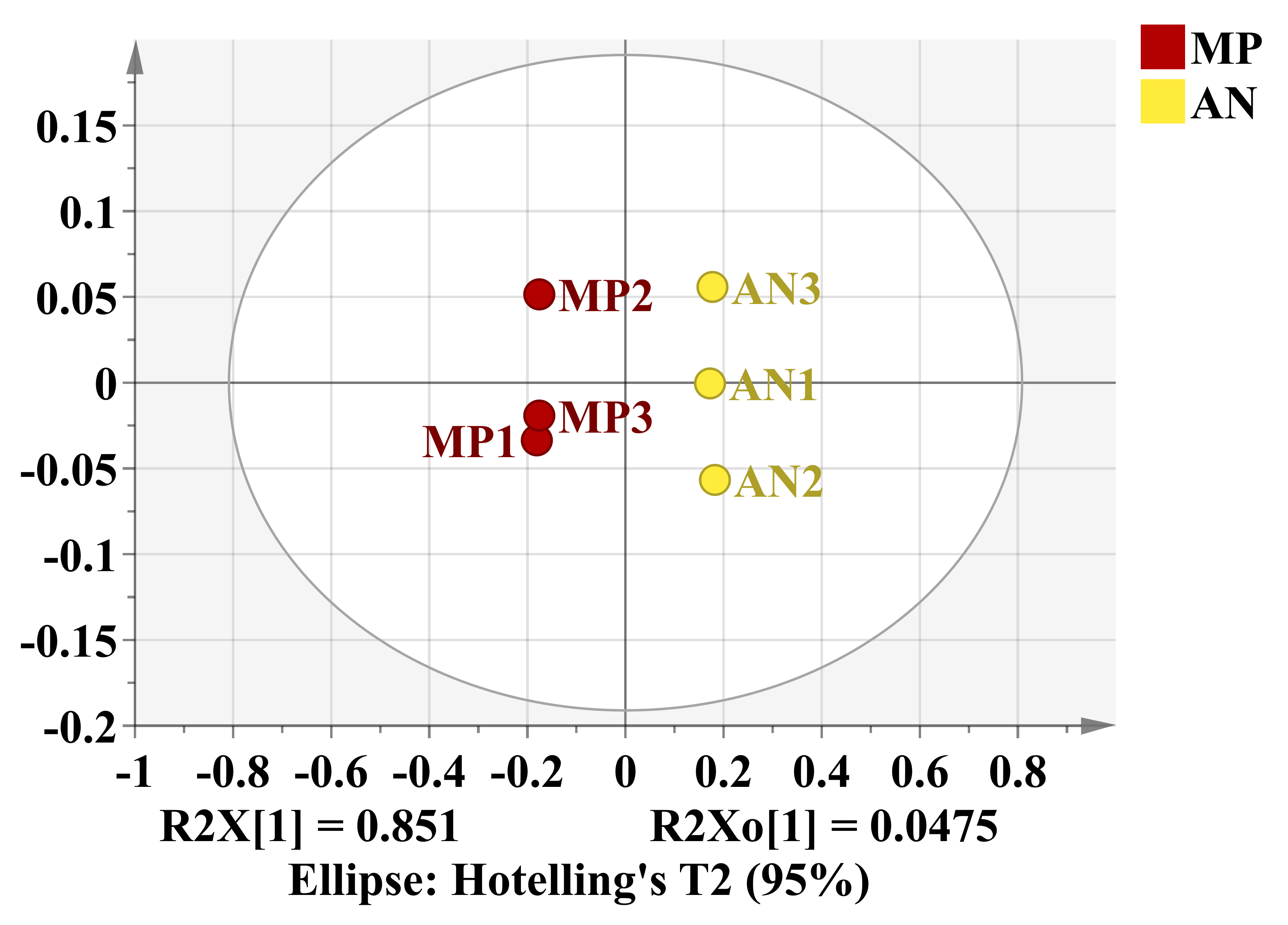 | 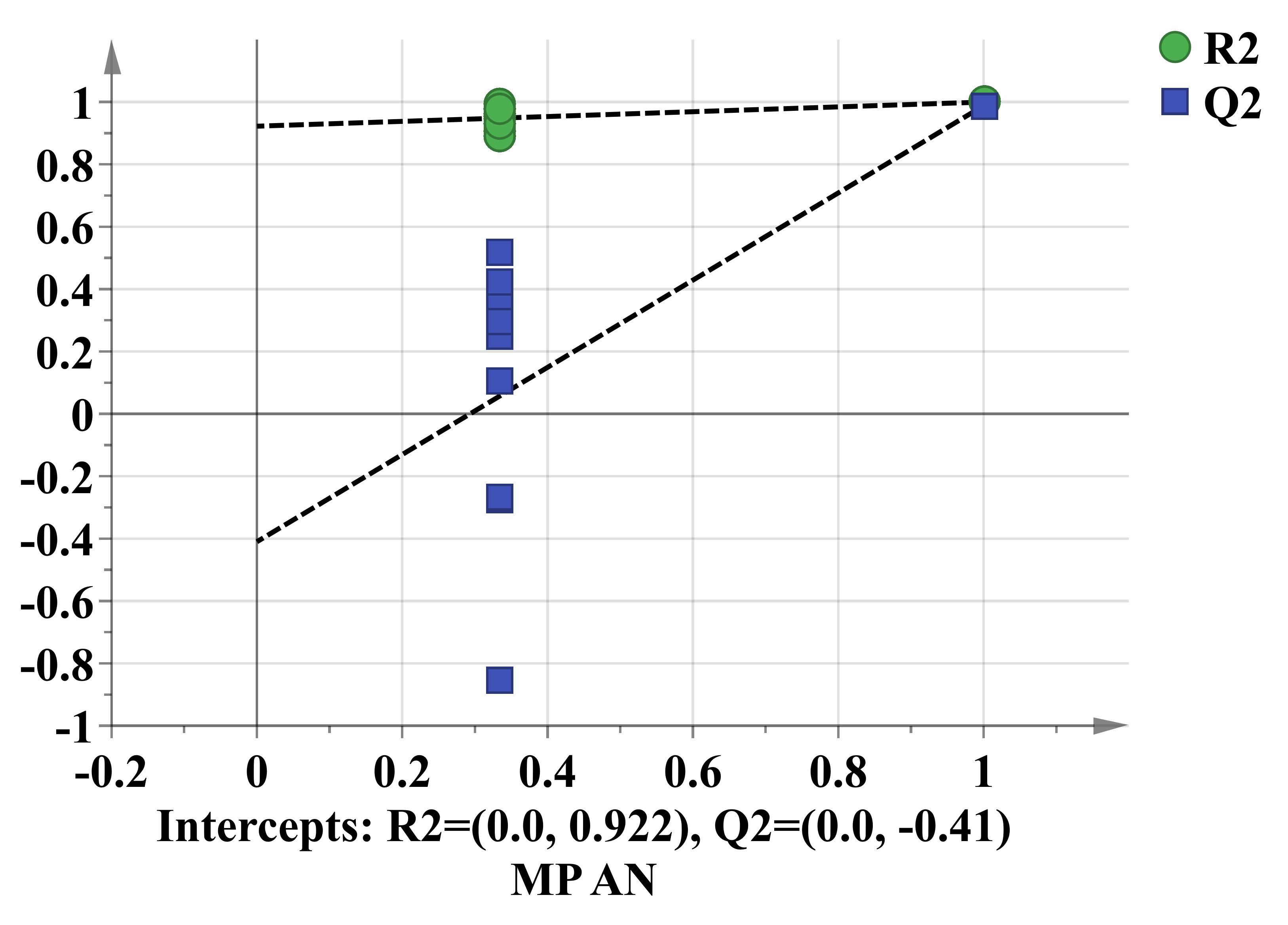 |

Fig. S1. Scoring plots and validation models for two-by-two comparison of the four famous freshwater fish in China.

**Note:** CI: *Ctenopharyngodon Idella*, HM: *Hypophthalmichthys molitrix*, MP: *Mylopharyngodon piceus*, AN: *Aristichthys nobilis*.


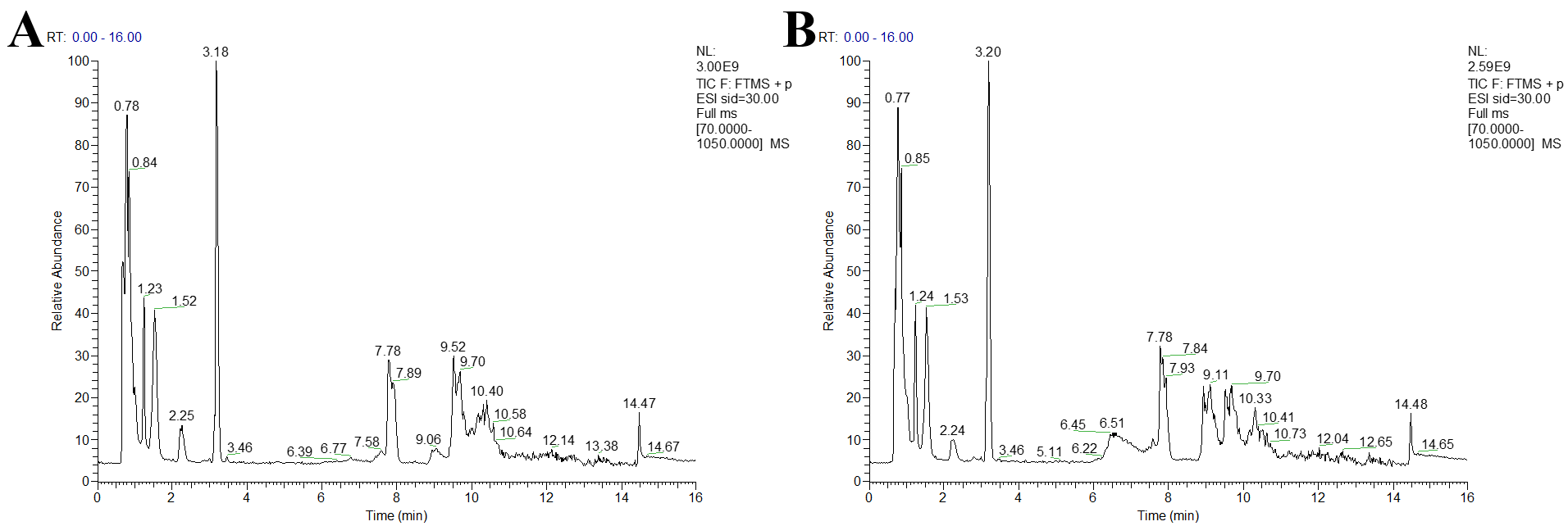

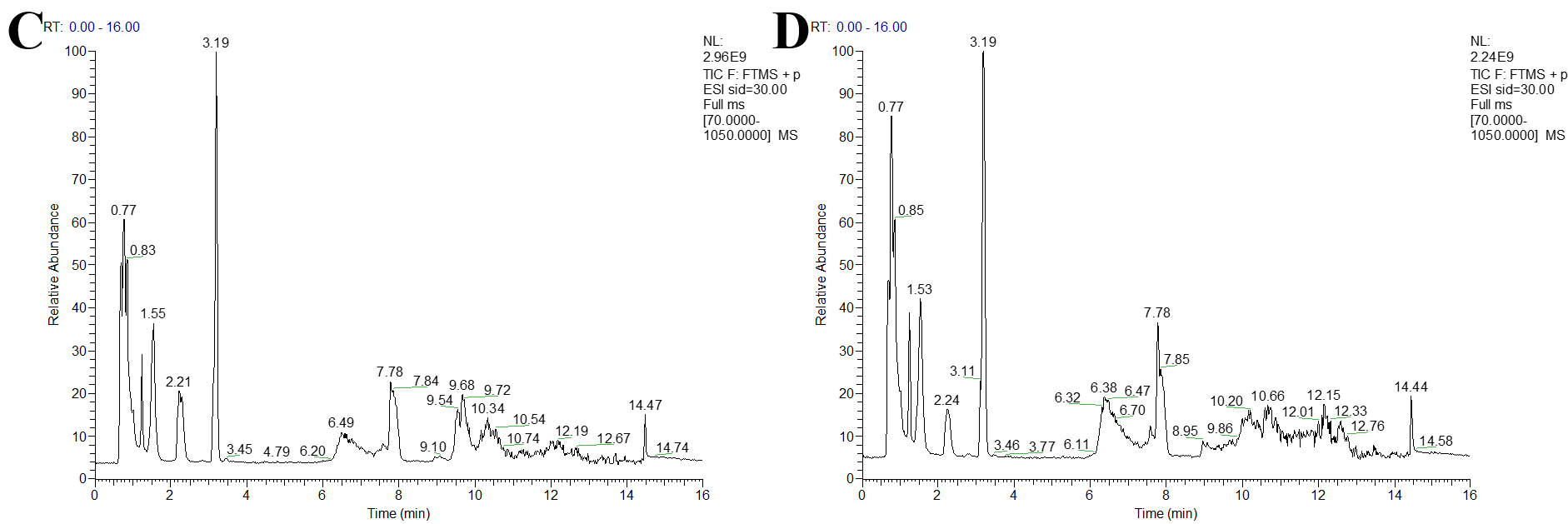


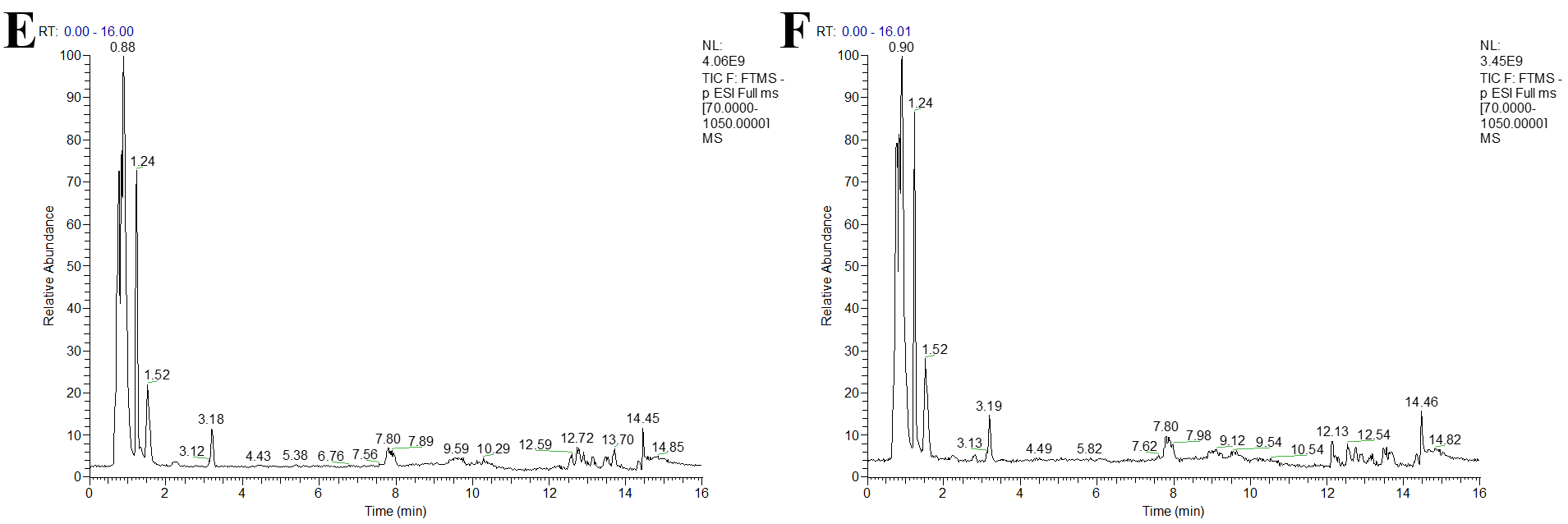


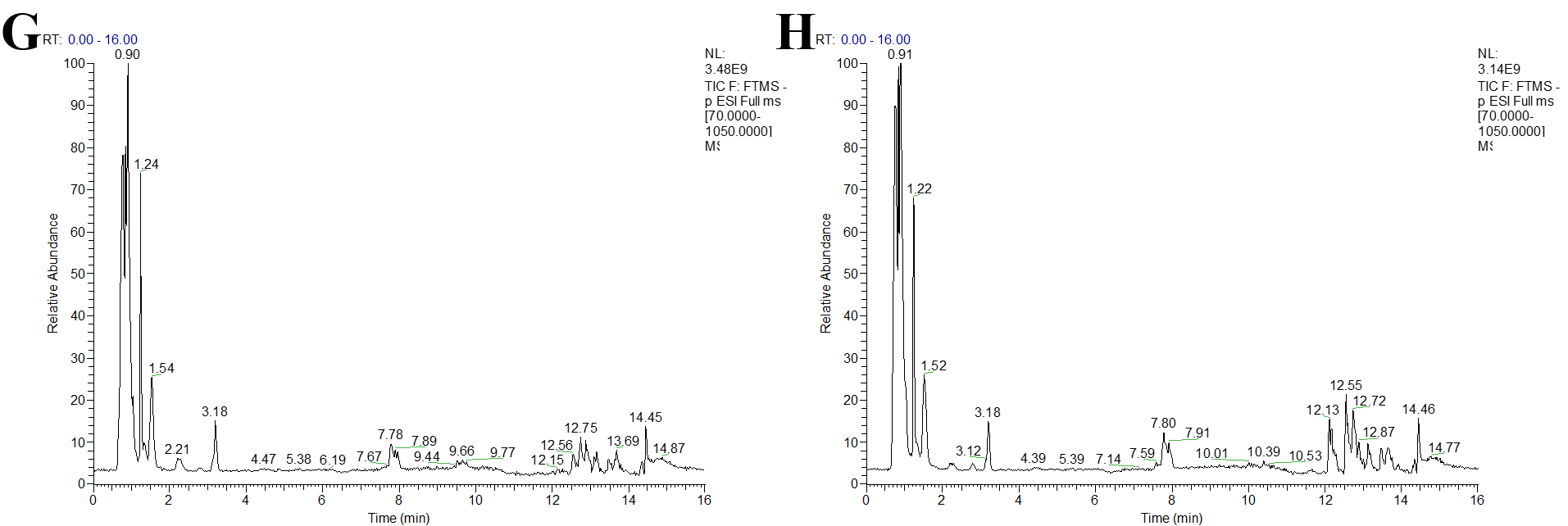


Fig. S2 The total ion characterization (TIC) chromatogram of the four famous freshwater fish in China in ESI+ mode and ESI- mode. ESI+ mode of CI (A); HM (B); MP (C); AN (D);ESI- mode of CI (E); HM (F); MP (G); AN (H).

Note: CI: *Ctenopharyngodon Idella*, HM: *Hypophthalmichthys molitrix*, MP: *Mylopharyngodon piceus*, AN: *Aristichthys nobilis*.

Table S1 Response strength and the relative standard deviation (RSD) of electronic tongue sensors in relation to the four famous freshwater fish in China.

| **sensors** |  | **CI** | **HM** | **MP** | **AN** |
| --- | --- | --- | --- | --- | --- |
| **AHS** | mean± SD | 5520.86±5.20 | 5984.32±11.36 | 5667.82±16.64 | 5921.79±10.30 |
|  | RSD (%) | 0.09 | 0.19 | 0.29 | 0.17 |
| **PKS** | mean± SD | 2151.37±3.74 | 2153.32±4.83 | 1929.07±6.11 | 2196.41±3.66 |
|  | RSD (%) | 0.17 | 0.22 | 0.32 | 0.17 |
| **CTS** | mean± SD | 3021.60±1.28 | 2918.63±1.99 | 2943.95±5.44 | 2949.77±1.58 |
|  | RSD (%) | 0.04 | 0.07 | 0.19 | 0.05 |
| **NMS** | mean± SD | 3126.90±6.23 | 3180.62±5.09 | 2968.75±5.07 | 3173.27±4.31 |
|  | RSD (%) | 0.20 | 0.16 | 0.17 | 0.14 |
| **CPS** | mean± SD | 2452.94±4.18 | 2443.32±2.42 | 2518.22±17.84 | 2449.16±1.03 |
|  | RSD (%) | 0.17 | 0.10 | 0.71 | 0.04 |
| **ANS** | mean± SD | 3548.97±4.12 | 3633.27±0.43 | 3556.96±1.28 | 3651.66±1.28 |
|  | RSD (%) | 0.12 | 0.01 | 0.04 | 0.03 |
| **SCS** | mean± SD | 5814.47±2.65 | 6252.85±10.58 | 6004.22±8.65 | 6197.70±8.54 |
|  | RSD (%) | 0.05 | 0.17 | 0.14 | 0.14 |

**Note:** CI: *Ctenopharyngodon Idella*, HM: *Hypophthalmichthys molitrix*, MP: *Mylopharyngodon piceus*, AN: *Aristichthys nobilis*.

Table S2 Parameters of OPLS-DA models for comparison of the four famous freshwater fish in China metabolites in the ESI+ and ESI- modes.

|  | R2X | R2Y | Q2 |
| --- | --- | --- | --- |
| ESI+ | | | |
| CI vs. HM | 0.88 | 0.997 | 0.981 |
| CI vs. MP | 0.867 | 1 | 0.995 |
| CI vs. AN | 0.876 | 0.999 | 0.995 |
| HM vs. MP | 0.898 | 1 | 0.997 |
| HM vs. AN | 0.908 | 1 | 0.998 |
| MP vs. AN | 0.911 | 1 | 0.996 |
| ESI- | | | |
| CI vs. HM | 0.872 | 0.999 | 0.99 |
| CI vs. MP | 0.796 | 0.999 | 0.952 |
| CI vs. AN | 0.865 | 0.999 | 0.991 |
| HM vs. MP | 0.866 | 0.999 | 0.994 |
| HM vs. AN | 0.927 | 0.997 | 0.991 |
| MP vs. AN | 0.898 | 1 | 0.988 |

**Note:** CI: *Ctenopharyngodon Idella*, HM: *Hypophthalmichthys molitrix*, MP: *Mylopharyngodon piceus*, AN: *Aristichthys nobilis*.

Table S3 Metabolic pathway of key metabolites in the four famous freshwater fish in China

| Metabolite | KEGG pathway ID | KEGG pathway name |
| --- | --- | --- |
| Choline | Map 00260, map 00564 | Glycine, serine and threonine metabolism; Glycerophospholipid metabolism |
| Acetylcholine | Map 00564 | Glycerophospholipid metabolism |
| palmitoylcarnitine | Map 00071 | Fatty acid degradation |
| linoleic acid | Map 00591 | Linoleic acid metabolism |
| arachidonic acid |  |  |
| pantothenic acid | Map 00410 | beta-Alanine metabolism |
| pipecolinic acid | Map 00310 | Lysine degradation |
| L-Glutathione | Map 00480 | Glutathione metabolism |

**Table S4 Profile of key metabolites in the four major Chinese carps**

| **Class** |  | **Name** | **VIP** | **Formular** | **m/z** | **RT/min** | **reference ion** | **CI** | **HM** | **MP** | **AN** |
| --- | --- | --- | --- | --- | --- | --- | --- | --- | --- | --- | --- |
| Lipids and analogues | FAs | Retinyl acetate | 9.21403 | C22 H32 O2 | 327.233 | 12.552 | [M-H]-1 | 3.61E-03±2.18E-04^c^ | 4.90E-03±1.16E-04^bc^ | 5.73E-03±2.64E-04^b^ | 1.47E-02±1.29E-03^a^ |
| Lipids and analogues | GLs | 1-(1Z-hexadecenyl)-sn-glycero-3-phosphocholine | 8.35707 | C24 H50 N O6 P | 480.3446 | 10.645 | [M+H]+1 | 1.19E-03±1.36E-04^b^ | 1.03E-03±1.36E-04^b^ | 6.29E-04±6.66E-05^b^ | 9.15E-03±6.10E-04^a^ |
| Lipids and analogues | PLs | LysoPC(22:5(7Z,10Z,13Z,16Z,19Z)) | 6.85995 | C30 H52 N O7 P | 570.3547 | 10.286 | [M+H]+1 | 6.65E-03±2.61E-04^a^ | 5.29E-03±5.09E-04^b^ | 6.13E-03±1.47E-04^a^ | 6.33E-04±2.30E-05^c^ |
| Lipids and analogues | GLs | 1-Oleoyl-sn-glycero-3-phosphocholine | 6.84368 | C26 H52 N O7 P | 522.3552 | 10.551 | [M+H]+1 | 7.51E-03±1.71E-03^a^ | 6.31E-03±3.12E-04^a^ | 7.05E-03±6.49E-04^a^ | 1.25E-03±5.96E-05^b^ |
| Lipids and analogues | GLs | 1-Linoleoyl-2-Hydroxy-sn-glycero-3-PC | 5.42722 | C26 H50 N O7 P | 520.3395 | 9.685 | [M+H]+1 | 8.02E-03±1.36E-03^a^ | 1.71E-03±1.08E-04^c^ | 3.82E-03±1.85E-04^b^ | 2.02E-04±2.84E-05^d^ |
| Lipids and analogues | PLs | Choline | 4.76834 | C5 H13 N O | 104.1074 | 10.631 | [M+H]+1 | 1.44E-03±1.97E-04^b^ | 1.51E-03±1.08E-04^b^ | 1.56E-03±7.99E-05^b^ | 4.18E-03±2.29E-04^a^ |
| Lipids and analogues | GLs | 1-Palmitoyl-sn-glycero-3-phosphocholine | 4.60594 | C24 H50 N O7 P | 496.3393 | 10.192 | [M+H]+1 | 5.49E-03±7.11E-04^b^ | 3.09E-03±2.28E-04^c^ | 1.78E-03±1.01E-04^d^ | 7.06E-03±1.44E-04^a^ |
| Lipids and analogues | GLs | 1-pentadecanoyl-2-hydroxy-sn-glycero-3-phosphoethanolamine | 3.96539 | C20 H42 N O7 P | 440.2765 | 7.802 | [M+H]+1 | 2.03E-02±9.14E-04^a^ | 1.98E-02±7.60E-04^a^ | 1.71E-02±3.55E-04^b^ | 1.62E-02±6.67E-04^b^ |
| Lipids and analogues | FAs | Ethyl eicosapentaenoic acid | 3.31603 | C22 H34 O2 | 329.2486 | 13.122 | [M-H]-1 | 3.05E-03±2.63E-04^b^ | 1.66E-03±1.33E-04^c^ | 3.17E-03±1.15E-04^b^ | 4.54E-03±2.04E-04^a^ |
| Lipids and analogues | GLs | Glycerophosphorylethanolamine | 2.96873 | C5 H14 N O6 P | 214.0481 | 0.761 | [M-H]-1 | 1.71E-03±9.92E-05^b^ | 1.01E-03±4.72E-05^d^ | 1.34E-03±4.07E-05^c^ | 2.62E-03±1.05E-04^a^ |
| Lipids and analogues | FAs | (+/-)3-Epi CP 47,497-C8-homolog | 2.90411 | C22 H36 O2 | 331.2643 | 13.475 | [M-H]-1 | 3.25E-04±1.50E-05^b^ | 3.47E-04±2.02E-05^b^ | 3.15E-04±2.14E-05^b^ | 1.31E-03±2.24E-05^a^ |
| Lipids and analogues | FAs | Bardoxolone methyl | 2.87709 | C32 H43 N O4 | 564.3309 | 9.673 | [M-H+HAc]-1 | 1.80E-03±6.95E-05^a^ | 3.72E-04±2.70E-05^c^ | 1.44E-03±1.67E-04^b^ | 8.22E-05±6.00E-06^d^ |
| Lipids and analogues | PLs | LysoPC(20:5(5Z,8Z,11Z,14Z,17Z)) | 2.86805 | C28 H48 N O7 P | 542.3237 | 9.071 | [M+H]+1 | 1.17E-03±3.37E-05^b^ | 1.61E-02±1.65E-03^a^ | 5.45E-04±3.24E-05^b^ | 3.81E-04±2.21E-05^b^ |
| Lipids and analogues | PLs | Acetylcholine | 2.86073 | C7 H15 N O2 | 146.1176 | 0.792 | [M+H]+1 | 7.31E-04±5.48E-05^c^ | 2.50E-03±1.38E-04^b^ | 2.37E-03±6.87E-05^b^ | 3.12E-03±3.17E-05^a^ |
| Lipids and analogues | PLs | LysoPC(P-18:0) | 2.80249 | C26 H54 N O6 P | 508.3762 | 11.125 | [M+H]+1 | 2.76E-04±4.65E-05^b^ | 9.88E-05±7.16E-06^b^ | 4.53E-05±1.76E-05^b^ | 1.22E-03±5.48E-04^a^ |
| Lipids and analogues | FAs | Kinoprene | 2.09968 | C18 H28 O2 | 275.2017 | 11.679 | [M-H]-1 | 2.28E-05±2.96E-06^c^ | 3.18E-04±2.18E-05^b^ | 2.28E-05±1.19E-05^c^ | 6.38E-04±3.32E-05^a^ |
| Lipids and analogues | PLs | 2-C-methylerythritol 4-phosphate | 2.08853 | C5 H13 O7 P | 215.0323 | 0.775 | [M-H]-1 | 1.27E-03±1.72E-04^b^ | 1.97E-03±8.36E-05^a^ | 1.10E-03±1.64E-04^b^ | 5.83E-04±6.07E-05^c^ |
| Lipids and analogues | GLs | 1-[(8Z,11Z,14Z)-icosatrienoyl]-sn-glycero-3-phosphocholine | 2.06741 | C28 H52 N O7 P | 546.3551 | 9.996 | [M+H]+1 | 1.22E-03±1.86E-04^a^ | 1.64E-04±1.70E-05^c^ | 6.11E-04±2.66E-05^b^ | 3.03E-05±8.74E-06^c^ |
| Lipids and analogues | PLs | LysoPC(22:4(7Z,10Z,13Z,16Z)) | 2.05321 | C30 H54 N O7 P | 572.3705 | 10.689 | [M+H]+1 | 8.30E-04±5.05E-05^a^ | 4.79E-04±3.34E-05^b^ | 4.14E-04±2.76E-05^b^ | 3.17E-05±3.20E-06^c^ |
| Lipids and analogues | GLs | 1-[(9Z)-hexadecenoyl]-sn-glycero-3-phosphocholine | 1.95483 | C24 H48 N O7 P | 494.3238 | 9.277 | [M+H]+1 | 1.24E-03±1.51E-04^a^ | 1.40E-03±9.45E-05^a^ | 2.22E-04±8.43E-06^b^ | 1.27E-04±1.00E-05^b^ |
| Lipids and analogues | PLs | Palmitoylcarnitine | 1.93669 | C23 H45 N O4 | 400.3419 | 10.104 | [M+H]+1 | 8.24E-05±1.08E-06^b^ | 1.40E-05±4.42E-07^c^ | 3.07E-05±3.08E-06^c^ | 4.98E-04±1.69E-05^a^ |
| Lipids and analogues | FAs | Isopropyl myristate | 1.63175 | C17 H34 O2 | 269.2486 | 14.141 | [M-H]-1 | 6.25E-05±6.48E-06^c^ | 1.81E-04±4.84E-06^b^ | 6.14E-05±6.81E-06^c^ | 4.06E-04±2.33E-05^a^ |
| Lipids and analogues | FAs | Methyl (9E)-9-octadecenoate | 1.60785 | C19 H36 O2 | 295.2642 | 14.302 | [M-H]-1 | 5.31E-05±8.05E-06^c^ | 9.68E-05±2.39E-05^b^ | 4.31E-05±9.27E-06^c^ | 3.61E-04±2.43E-05^a^ |
| Organic acids |  | cis-5,8,11,14,17-Eicosapentaenoic acid | 9.9048 | C20 H30 O2 | 301.2173 | 12.136 | [M-H]-1 | 1.22E-03±6.29E-05^c^ | 7.32E-03±6.90E-04^b^ | 9.44E-04±6.62E-05^c^ | 1.47E-02±1.28E-03^a^ |
| Organic acids |  | Arachidonic acid | 6.80465 | C20 H32 O2 | 303.2329 | 12.743 | [M-H]-1 | 8.12E-03±8.15E-04^b^ | 4.92E-03±1.34E-04^c^ | 8.00E-03±2.10E-04^b^ | 1.37E-02±5.90E-04^a^ |
| Organic acids |  | Alpha-Linolenic acid | 3.16792 | C18 H30 O2 | 277.2172 | 12.247 | [M-H]-1 | 5.71E-04±4.63E-05^c^ | 1.25E-03±9.03E-06^b^ | 7.39E-04±2.16E-04^c^ | 2.04E-03±1.16E-04^a^ |
| Organic acids |  | Oleic acid | 2.4509 | C18 H34 O2 | 281.2485 | 13.678 | [M-H]-1 | 3.45E-03±1.40E-04^b^ | 2.94E-03±9.32E-05^b^ | 4.38E-03±4.38E-04^a^ | 4.84E-03±6.39E-04^a^ |
| Organic acids |  | Pantothenic acid | 2.25456 | C9 H17 N O5 | 218.103 | 2.79 | [M-H]-1 | 4.36E-04±5.64E-06^d^ | 1.39E-03±9.99E-05^b^ | 1.07E-03±8.09E-05^c^ | 1.67E-03±7.19E-05^a^ |
| Organic acids |  | D-(+)-Pipecolinic acid | 1.99523 | C6 H11 N O2 | 130.0864 | 0.712 | [M+H]+1 | 2.36E-03±1.62E-04^a^ | 1.34E-04±7.99E-05^b^ | 1.15E-04±1.53E-05^b^ | 2.41E-05±5.91E-07^b^ |
| Organic acids |  | Palmitic Acid | 1.88745 | C16 H32 O2 | 255.2328 | 13.501 | [M-H]-1 | 1.60E-03±5.08E-05^d^ | 2.15E-03±7.87E-05^b^ | 1.78E-03±5.31E-05^c^ | 2.31E-03±4.96E-05^a^ |
| Organic acids |  | Linoleic acid | 1.67185 | C18 H32 O2 | 279.2328 | 12.908 | [M-H]-1 | 2.53E-03±2.29E-04^b^ | 1.55E-03±7.56E-05^c^ | 3.93E-03±3.25E-04^a^ | 1.99E-03±1.61E-04^c^ |
| Organic acids |  | 9E-heptadecenoic acid | 1.56771 | C17 H32 O2 | 267.233 | 13.155 | [M-H]-1 | 4.90E-05±1.04E-05^c^ | 1.57E-04±3.93E-06^b^ | 4.01E-05±1.18E-05^c^ | 3.68E-04±6.45E-05^a^ |
| Organic nitrogen compounds |  | 2-Amino-4-methylpyrimidine | 7.91588 | C5 H7 N3 | 110.0716 | 0.682 | [M+H]+1 | 3.19E-02±3.59E-03^a^ | 2.29E-02±4.54E-04^b^ | 3.21E-02±2.34E-03^a^ | 2.01E-02±1.00E-03^b^ |
| Organic nitrogen compounds |  | Fexofenadine | 3.23114 | C32 H39 N O4 | 500.2785 | 9.579 | [M-H]-1 | 1.76E-03±1.47E-04^a^ | 1.19E-03±1.04E-04^b^ | 1.73E-03±7.33E-05^a^ | 3.48E-04±2.53E-05^c^ |
| Organic nitrogen compounds |  | Allopurinol | 3.20602 | C5 H4 N4 O | 137.0458 | 1.536 | [M+H]+1 | 2.55E-02±1.81E-03^b^ | 3.17E-02±2.60E-03^a^ | 2.76E-02±1.16E-03^ab^ | 2.46E-02±1.50E-03^b^ |
| Organic nitrogen compounds |  | DL-Stachydrine | 2.51612 | C7 H13 N O2 | 144.1019 | 0.836 | [M+H]+1 | 2.24E-03±1.24E-04^a^ | 5.43E-04±5.33E-05^c^ | 9.75E-04±6.90E-05^b^ | 2.68E-04±5.45E-06^d^ |
| Organic nitrogen compounds |  | 2-Aminoethyl (2R)-3-[(1Z)-1-hexadecen-1-yloxy]-2-hydroxypropyl hydrogen phosphate | 1.86806 | C21 H44 N O6 P | 438.2976 | 10.413 | [M+H]+1 | 1.49E-03±2.30E-04^a^ | 3.88E-04±3.63E-05^b^ | 3.90E-04±3.24E-05^b^ | 1.59E-03±1.37E-04^a^ |
| Organic nitrogen compounds |  | Nicotinamide | 1.80662 | C6 H6 N2 O | 123.0556 | 1.45 | [M+H]+1 | 2.88E-03±3.22E-04^a^ | 1.91E-03±1.08E-04^b^ | 1.42E-03±1.08E-04^b^ | 2.95E-03±4.40E-04^a^ |
| Amino acids and derivatives |  | L-Glutathione (reduced) | 2.38687 | C10 H17 N3 O6 S | 306.0766 | 0.841 | [M-H]-1 | 5.41E-03±5.88E-04^a^ | 6.39E-03±9.19E-04^a^ | 2.58E-03±1.04E-04^b^ | 2.99E-03±1.58E-05^b^ |
| Amino acids and derivatives |  | Cystathionine | 1.63775 | C7 H14 N2 O4 S | 221.0597 | 0.752 | [M-H]-1 | 1.49E-03±2.52E-04^a^ | 1.79E-04±1.36E-05^b^ | 2.52E-04±2.22E-05^b^ | 1.01E-04±6.25E-05^b^ |
| Amino acids and derivatives |  | C18-Carnitine | 1.61537 | C25 H49 N O4 | 428.3733 | 11.227 | [M+H]+1 | 1.61E-04±3.31E-05^b^ | 2.18E-05±5.37E-06^c^ | 3.74E-05±6.37E-06^c^ | 4.26E-04±3.26E-05^a^ |
| Amino acids and derivatives |  | L(-)-Carnitine | 1.50479 | C7 H15 N O3 | 162.1125 | 0.77 | [M+H]+1 | 5.35E-04±2.63E-05^c^ | 1.15E-03±7.82E-05^b^ | 1.16E-03±4.77E-05^b^ | 1.33E-03±2.73E-05^a^ |
| Nucleotide |  | Inosine-5'-monophosphate (IMP) | 3.45569 | C10 H13 N4 O8 P | 347.0397 | 0.829 | [M-H]-1 | 3.14E-02±2.67E-03^a^ | 1.65E-02±8.31E-03^b^ | 2.03E-02±1.67E-03^ab^ | 2.87E-02±2.50E-03^a^ |
| Nucleoside |  | Inosine | 1.84226 | C10 H12 N4 O5 | 267.0735 | 1.534 | [M-H]-1 | 2.09E-02±4.60E-04^b^ | 2.58E-02±5.93E-04^a^ | 2.60E-02±1.39E-03^a^ | 2.24E-02±2.22E-04^b^ |
| Other |  | Benzene | 3.15143 | C6 H6 | 120.0811 | 2.242 | [M+ACN+H]+1 | 8.80E-03±1.57E-04^b^ | 5.34E-03±2.73E-04^c^ | 2.19E-02±1.61E-03^a^ | 8.72E-03±4.20E-04^b^ |
| Other |  | D-(-)-Mannitol | 1.99656 | C6 H14 O6 | 217.0478 | 0.772 | [M+Cl]-1 | 2.22E-05±3.03E-06^b^ | 7.94E-05±4.23E-06^b^ | 5.00E-05±4.47E-06^b^ | 5.21E-04±8.37E-05^a^ |
| Other |  | 4-Hydroxybenzaldehyde | 1.64847 | C7 H6 O2 | 123.0443 | 1.244 | [M+H]+1 | 1.04E-03±2.59E-05^a^ | 5.33E-04±3.15E-05^c^ | 8.44E-04±3.74E-05^b^ | 4.37E-04±2.37E-05^d^ |
| Other |  | Androstanolone | 1.51721 | C19 H30 O2 | 289.2174 | 12.278 | [M-H]-1 | 4.13E-06±2.57E-07^c^ | 8.68E-05±4.36E-06^b^ | 4.89E-06±1.30E-06^c^ | 3.04E-04±6.28E-05^a^ |
| Other |  | Pyrophosphoric Acid | 2.02695 | H4 O7 P2 | 176.9351 | 0.957 | [M-H]-1 | 3.15E-03±6.69E-04^b^ | 3.80E-03±1.48E-04^ab^ | 3.66E-03±9.51E-05^ab^ | 4.27E-03±2.34E-04^a^ |

**Note:** The value is expressed as S.D. ± mean. Significant differences are represented by distinct letters within the same row (*p* < 0.05); CI: *Ctenopharyngodon Idella*, HM: *Hypophthalmichthys molitrix*, MP: *Mylopharyngodon piceus*, AN: *Aristichthys nobilis*.
